# Fluorescence Lifetime-Based FRET Biosensors for Monitoring N Terminal Domain-Dependent Interactions of TDP-43 in Living Cells: A Novel Approach for ALS and FTD Drug Discovery

**DOI:** 10.1021/acschemneuro.5c00266

**Published:** 2025-06-10

**Authors:** Noah Nathan Kochen, Marguerite Murray, Sophia Zafari, Nagamani Vunnam, Elly E. Liao, Lihsia Chen, Anthony R. Braun, Jonathan N. Sachs

**Affiliations:** 1 Department of Biomedical Engineering, University of Minnesota, Minneapolis, Minnesota 55455, United States; 2 Department of Genetics, Cell Biology and Development, University of Minnesota, Minneapolis, Minnesota 55455, United States

**Keywords:** TDP-43, ALS, FRET

## Abstract

Pathological aggregates of TDP-43 are implicated in Alzheimer’s disease, frontotemporal dementia, and amyotrophic lateral sclerosis. While therapeutic efforts have traditionally focused on mitigating end-stage TDP-43 aggregation, recent evidence highlights an upstream and potentially targetable event: the loss of functional nuclear TDP-43 multimers due to disrupted N-terminal domain (NTD) interactions. To address this, we developed fluorescence lifetime (FLT)-based FRET biosensors to monitor TDP-43 multimerization in living cells that couple a full-length TDP-43 FLT-FRET biosensor screen with an NTD-deletion counter screen, forming the foundation of a novel high-throughput screening (HTS) platform. Screening the 2682 compound FDA-approved Selleck library, we identified the small molecule ketoconazole, which stabilizes functional nuclear TDP-43 multimers in an NTD-dependent manner with low micromolar potency. Ketoconazole rescues TDP-43 mislocalization and aggregation, restores SREBP2 mRNA levels under TDP-43 overexpression, improves neuronal health, and partially restores motor function in a TDP-43 model. These findings establish both the biosensors and the HTS platform as innovative tools for TDP-43 drug discovery and support an exciting translational approach for targeting TDP-43 proteinopathies.

## Graphical abstract

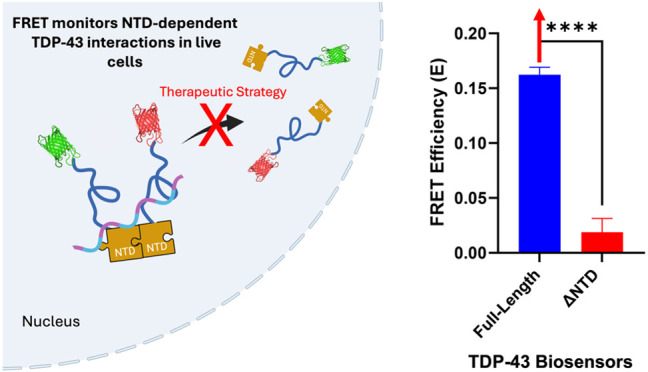

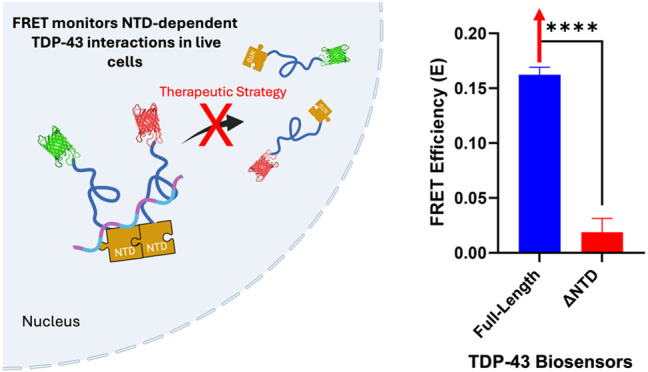

## Introduction

TAR DNA-binding protein 43 (TDP-43) proteinopathies are present in roughly 75% of Alzheimer’s disease (AD), 97% of amyotrophic lateral sclerosis (ALS) and 50% of frontotemporal dementia (FTD) cases, spanning both sporadic and familial forms of these diseases.^1^ The pathophysiology of these proteinopathies involves both TDP-43 gain of toxic functions (e.g., neuroinflammation, mitochondrial dysfunction and proteostatic stress driven by mislocalization and aggregation of TDP-43) and loss of native functions (e.g., loss of RNA-processing, reversible stress granule formation and axonal transport).^2,3^ Due to the widespread presence of TDP-43 cytoplasmic inclusions in late-stage ALS and FTD,^4^ numerous small molecule screening strategies have focused on inhibiting its pathological aggregation.^5–8^

TDP-43 is a multidomain protein involved in RNA stability, splicing and transport as well as formation of cytosolic stress granules during cellular stress.^9^ TDP-43 contains a globular N-terminal domain (NTD) involved in its native self-oligomerization into dimers and multimers, two globular RNA recognition motifs (RRMs) and a glycine-rich disordered C-terminal domain (CTD) implicated in the formation of amyloid aggregates.^10^ Assemblies formed via CTD-CTD interactions are important for formation of cytoplasmic stress granules in response to cellular stress; however, if unresolved, these assemblies can lead to irreversible aggregation linked to TDP-43’s toxic gain of function pathology.^11,12^

Notably, not all TDP-43 assemblies are detrimental to cellular health. TDP-43’s nuclear retention and native functions — RNA-binding and splicing activity — require oligomerization mediated by NTD-NTD interactions.^13–15^ Furthermore, eliminating NTD-mediated dimer/multimers via point mutations renders TDP-43 more susceptible to mislocalization, aggregation, and reduces its resident cellular half-life.^16^ A recent seminal study by Oiwa et al. identified a significant loss of functional NTD-mediated TDP-43 dimers/multimers in the brains and spinal cords of ALS patients.^15^ This pathological decrease in TDP-43 multimerization precedes toxic gain of function, namely TDP-43 mislocalization to the cytoplasm, subsequent unresolved aggregation, and hyperphosphorylation.^17–23^

This study builds upon these findings through the development of a novel therapeutic discovery platform that monitors NTD-dependent interactions of nuclear TDP-43 in living cells. We have engineered a series of live-cell, fluorescence lifetime (FLT) based Förster resonance energy transfer (FRET) biosensors that monitor full-length (FL) and NTD-null (ΔNTD) TDP-43 homo-oligomerization. In conjunction, the FL and ΔNTD FRET biosensors allow us to discriminate between NTD-dependent and NTD-independent interactions, as native, nonpathological TDP-43 self-interactions are almost completely abolished with NTD deletion. Our previous work employed a similar FLT-FRET strategy for high-throughput screening (HTS) campaigns targeting other neurodegenerative disease associated proteins (e.g., alpha-synuclein, tau, and huntingtin) which, similar to TDP-43, populate an ensemble of aggregation and native conformational states.^24–27^

This approach improves upon previously established TDP-43 bifluorescence complementation (BiFC) assays by enabling time-resolved, dynamic, and reversible measurements.^28^ While BiFC excels at monitoring protein assembly, it relies on irreversible complementation of split constructs and fluorophore maturation, limiting its ability to detect the disruption of pre-existing protein–protein interactions.^29^ In contrast, FLT-FRET is sensitive to the distance and orientation between donor and acceptor fluorophores, allowing for real-time monitoring of both the formation and disruption of interactions within living cells.^29^ Other methods, such as the split-luciferase NanoBiT assay, can monitor assembly and disassembly but rely on intensity-based measurements, which are susceptible to variability from differences in cell dispensing, construct expression, and substrate addition.^30,31^ FLT-FRET, being based on the intrinsic FLT of the fluorophore, overcomes these challenges, offering higher sensitivity and reliability for HTS applications.^32^

Using these TDP-43 biosensors we screened the 2,684-compound FDA-approved Selleck library, and the hit compound ketoconazole – a known P450 cytochrome inhibitor – is shown to increase NTD-dependent TDP-43 FRET and rescue a series of TDP-43 proteinopathy phenotypes in both cellular and *in vivo* models. The biosensor design and counter-screening strategy allows us to identify compounds that bias the TDP-43 ensemble toward nuclear NTD-dependent interactions. The identification of an NTD-dependent small molecule capable of rescuing TDP-43-induced deficits demonstrates that this platform is a novel and exciting therapeutic discovery platform for FTD, ALS, and other TDP-43 proteinopathies.

## Results

### Intermolecular Biosensor Monitors NTD-Dependent TDP-43 FLT-FRET

Biosensor constructs were engineered using a C-terminal fusion of either donor (mNeonGreen, mNg) or acceptor (mCherry, mCh) fluorescent proteins to full-length (FL) or NTD deletion (ΔNTD, removing residues 2–75, roughly ∼ 8 kDa smaller than FL TDP-43) human TDP-43 (Figure 1A). The use of the C-terminal fusion strategy maintains a consistent fluorophore placement across both the FL and ΔNTD biosensors. In addition, our focus is on monitoring NTD-dependent interactions and the C-terminal fusion reduces the potential for steric hindrance near the NTD domain. The mNg/mCh FRET pair provides an advantage over GFP/RFP biosensors due to the fluorophores’ high donor quantum yield (nearly three times as bright).^33^ In addition to TDP-43 biosensors, control donor-only mNg and donor–acceptor mCh-Linker-mNg (using a 28-residue G_4_S linker) biosensors were established.

**Figure 1:**
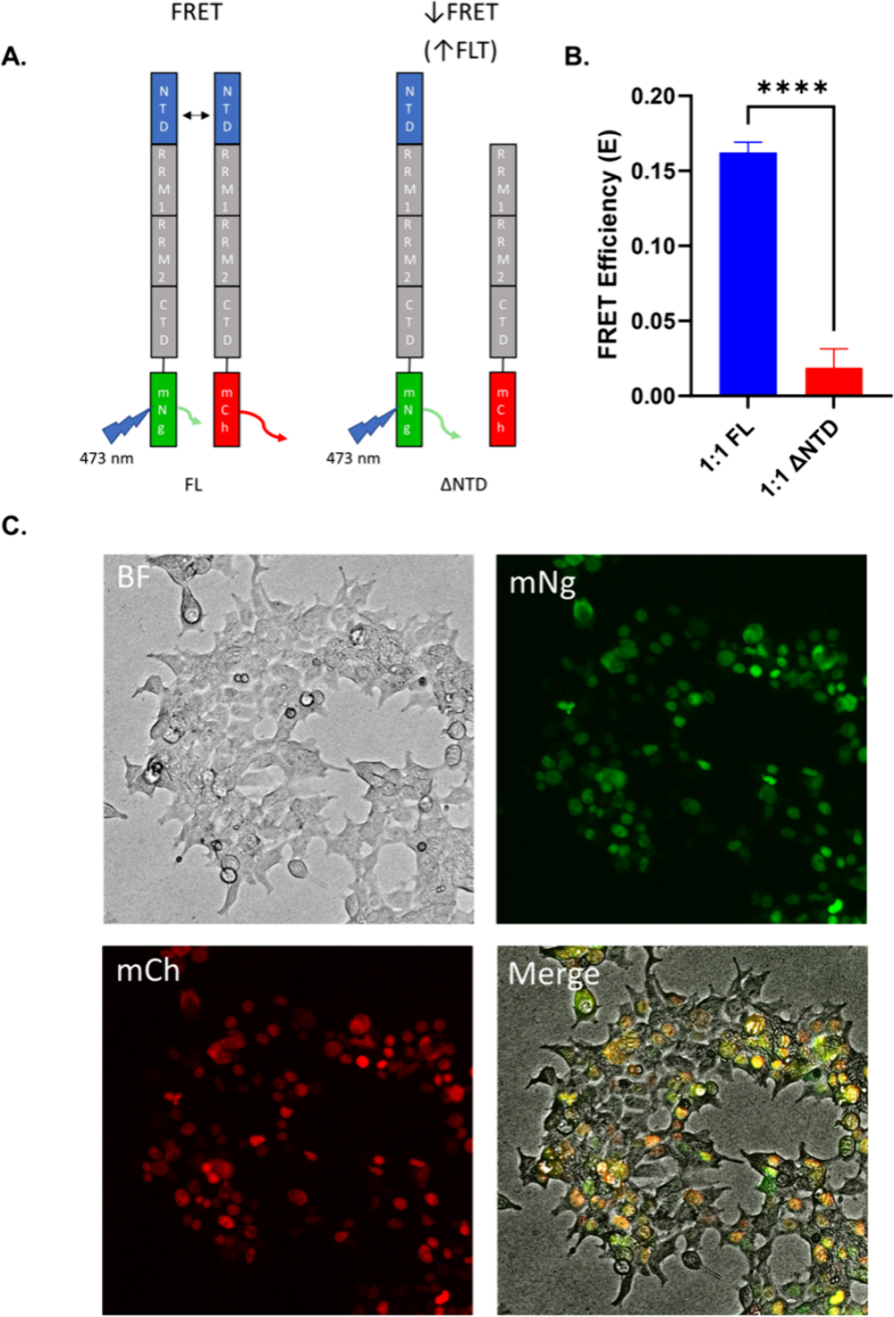
FLT-FRET TDP-43 biosensor monitors nuclear NTD-dependent TDP-43 interactions. (A) Diagrams of full-length (WT) and NTD-null (ΔNTD) TDP-43-fluorophore fusion biosensors. (B) FRET of FL and ΔNTD biosensors under 1:1 donor:acceptor ratio in HEK293T cells after 24 h of expression. (C) Fluorescence live-cell imaging of 1:1 FL biosensor expressed for 24 h in HEK293T cells. Statistic shown is a one-way ANOVA comparison with Bonferroni correction (subset of full donor:acceptor ratio experiment shown in Figure S2, ****p < 0.0001).

Biosensor FRET is measured by monitoring the donor’s FLT with and without expression of the acceptor (τ_DA_ and τ_D_, respectively), where FRET efficiency is E = 1-τ_DA_/τ_D_. Because FLT is an intrinsic property of the fluorophore, it provides increased sensitivity relative to intensity-based approaches previously utilized for monitoring TDP-43 oligomerization.^15,34,35^ Figure S1 illustrates the ∼ 16-fold reduction in variability for FLT versus fluorescence intensity in a high-density 1,536-well plate format — which allows for robust compound detection with high-throughput capacity — for the TDP-43 and linker biosensors.

Figure 1B shows that expression of the FL TDP-43-mNg + FL TDP-43-mCh at 1:1 donor to acceptor ratio results in a significantly higher FRET efficiency (E = 0.16 ± 0.0065) compared to the FL TDP-43-mNg + ΔNTD TDP-43-mCh biosensor (E = 0.02 ± 0.012). The FRET efficiency from the ΔNTD system is at similar levels observed for noninteracting background FRET biosensors used in our previous studies.^24,25^ Consistent with the TDP-43 BiFC measurements reported by Afroz et al., our FRET analysis reveals that, under basal conditions, TDP-43 self-interactions are predominantly mediated by the NTD.^35^ Interestingly, when total donor construct is held constant with increasing amounts of acceptor — increasing the total amount of TDP-43 — we observe a concomitant increase in FRET for both the FL and ΔNTD biosensors, suggesting the ensemble of TDP-43 assemblies is being biased toward non-NTD dependent species at higher levels of TDP-43 expression (Figure S2).

Although increasing the total amount of TDP-43 results in elevated FL TDP-43 FRET (i.e., a larger FRET signal window), the concomitant increase in NTD-independent FRET complicates the specific targeting of NTD-dependent interactions. Hence, all subsequent TDP-43 FRET assays are performed using the 1:1 biosensor condition. Expression of FL biosensors at this ratio results in diffuse nuclear localization of TDP-43 confirmed via Hoechst staining, devoid of puncta and cytoplasmic mislocalization (Figure 1C and Figure S3). Biosensor expression was confirmed via immunoblot analysis, which are described in detail in Figure S4.

Next, we evaluated both the FL and ΔNTD TDP-43 biosensor’s ability to form TDP-43 multimers using a disuccinimidyl glutarate (DSG) cross-linking protocol. HEK293T cells transiently transfected with FL or ΔNTD TDP-43-mNg constructs were harvested, cross-linked, and analyzed via Western blot. As expected, DSG cross-linking resulted in dimeric and multimeric TDP-43 bands across all conditions due to endogenous TDP-43 expression (Figure S5A). The addition of FL TDP-43-mNg resulted in the highest amount of cross-linked TDP-43 dimer/multimers, with higher molecular weight (HMW) bands comprising of TDP-43 fusion protein assemblies. In contrast, the ΔNTD TDP-43 biosensor expressing cells show only endogenous TDP-43 dimer/multimer bands. Densitometry analysis of the multimeric TDP-43 bands relative to total protein staining (Figure S5B-C and Figure S6) confirms these observations. Fusion of TDP-43 with mCherry resulted in the same profiles observed for mNeonGreen tagging shown in Figure S5A (Figure S7 and S8). The HMW TDP-43 band was present in the uncross-linked TDP-43-mNg and TDP-43-mCh condition, and to rule this out as an artifact of the XFP fusion, we show that unlabeled TDP-43 overexpression results in this same effect relative to untransfected and empty vector transfected cells (Figure S9). Taken together, the data suggests that the FL TDP-43 biosensor reports on nuclear NTD-dependent interactions in living cells.

### Selleck Library FLT-FRET HTS

Biosensor expression and plating consistency were monitored via coefficient of variation (CV) measurements in the 1536-well plate format. Figure 2A-B and Figure S1 confirm a robust FLT and FRET signal with low CV (∼1%) suitable for screening. It is important to note that FRET calculations include an error propagation due to the two separate FLT measurements (i.e., donor only, and donor + acceptor conditions). Therefore, for all HTS applications, hit compounds are identified via FLT change (ΔFLT) relative to DMSO-treated wells to improve the sensitivity of the screen.

**Figure 2:**
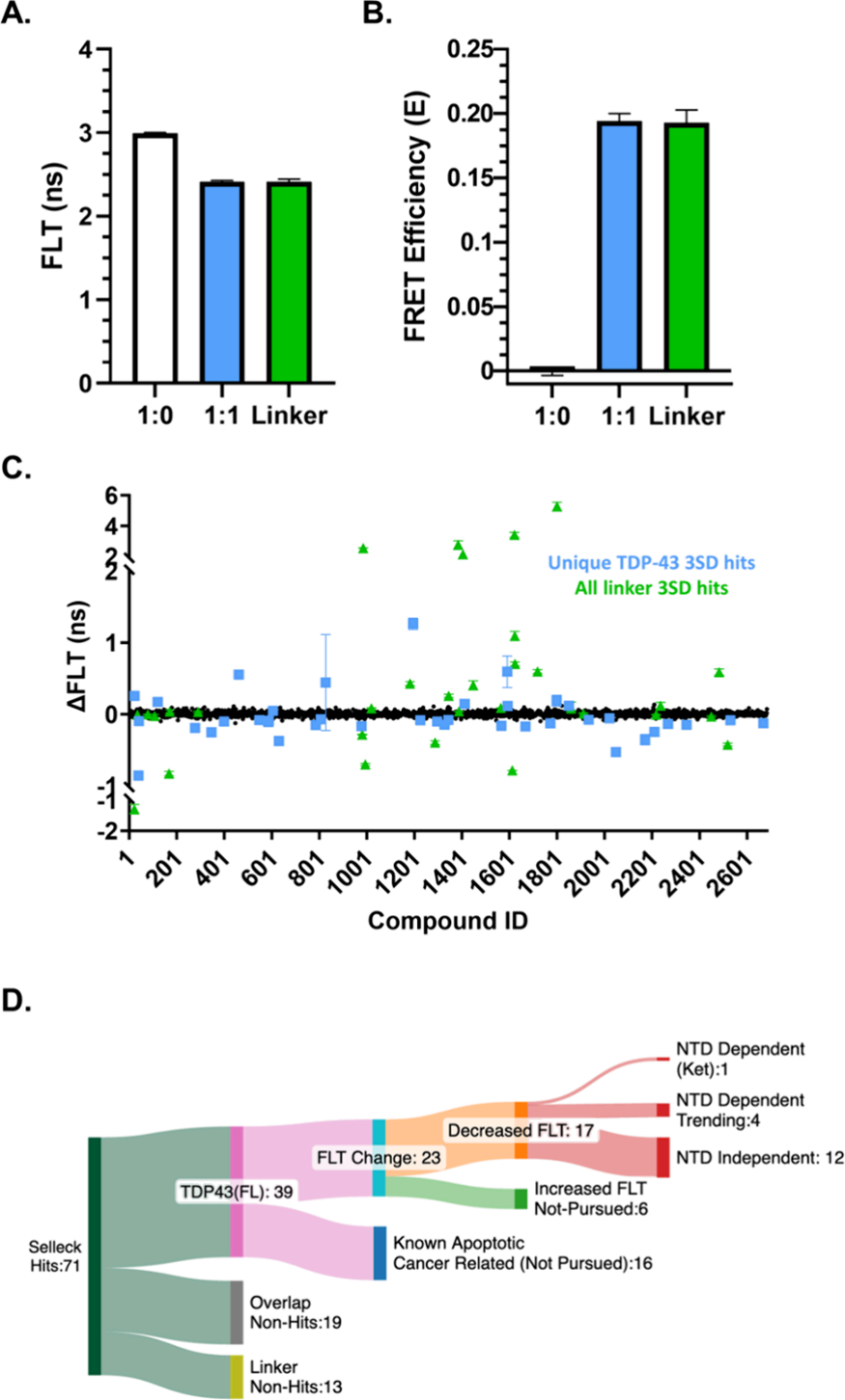
FDA-approved Selleck library screen in FL TDP-43 and linker control biosensors. (A) Average FLT of biosensors in HEK293T cells measured in 1536-well plate format for drug screening. (B) Average FRET efficiencies calculated from (A). (C) Average ΔFLT (FLT_drug_ – FLT_DMSO_) for FDA-approved Selleck library screened against FL and linker biosensors. Positive ΔFLT indicate molecules that decrease FRET, whereas negative ΔFLT indicate molecules that increase FRET. (D) Sankey diagram summary of TDP-43 hit filtering rationale (created using SankeyMATIC). Data shown are mean ± SEM from *N* = 3 independent experiments.

Next, we executed an HTS of the FDA-approved, 2,684-compound Selleck library. Biosensor cells were plated into 1536-well drug plates with compounds at a final concentration of 10 μM for 2 h. We conducted triplicate screens using the FL TDP-43 biosensor and counter-screens using the linker-only control (diagrams of biosensors used in the HTS campaign shown in Figure S10), which are essential to flag non-TDP-43 specific hits (e.g., those that nonspecifically affect the XFPs). Compounds with a reproducible ΔFLT of ± 3 standard deviation (SD) across the triplicate screens that were not flagged in either the linker screen or the fluorescent interference spectral similarity filter^25^ were considered FL TDP-43 unique hits. Figure 2C–D shows TDP-43 screen summary data as ΔFLT, highlighting ± 3SD TDP-43 unique hits in light blue and molecules that were flagged as ± 3SD hits in the control linker screens in green. Overall, we identified 58 hits, of which 19 were flagged in the linker counter-screen. Of the 39 unique TDP-43 hits, we excluded 16 due to known mechanism of action (MOA) associated with cell death and toxicity, resulting in 23 compounds (summarized in Table S1).

### Identification of NTD-Specific Hit Compounds via ΔNTD Biosensor Counter-Screening

Next, using the ΔNTD biosensor, we implemented a counter-screen strategy to identify compounds that increase NTD-dependent interactions. An NTD-dependent compound will have a unique ΔFLT profile, specifically decreasing FLT (increasing FRET) for the FL biosensor without affecting the ΔNTD biosensor. A summary of the counter-screen results (performed in triplicate) for the 23 TDP-43 unique hits at 10 μM against the ΔNTD biosensor is reported in Figure S11. Hits were flagged as NTD-dependent if only FL and not ΔNTD showed a statistically significant difference relative to their respective biosensor’s DMSO condition. From these 23 compounds, ketoconazole emerged as the sole hit that reduced TDP-43 biosensor FLT and satisfied the FL versus ΔNTD hit flag (Figure 3A). Figure 3B illustrates a 7-point ketoconazole dose–response that resulted in an FLT EC_50_ of 1.2 μM. Although not strictly meeting the NTD-dependent hit criterion, there are additional compounds that displayed a similar trend in NTD-dependent response that potentially warrant further exploration. A discussion of these compounds is presented later in the manuscript.

**Figure 3:**
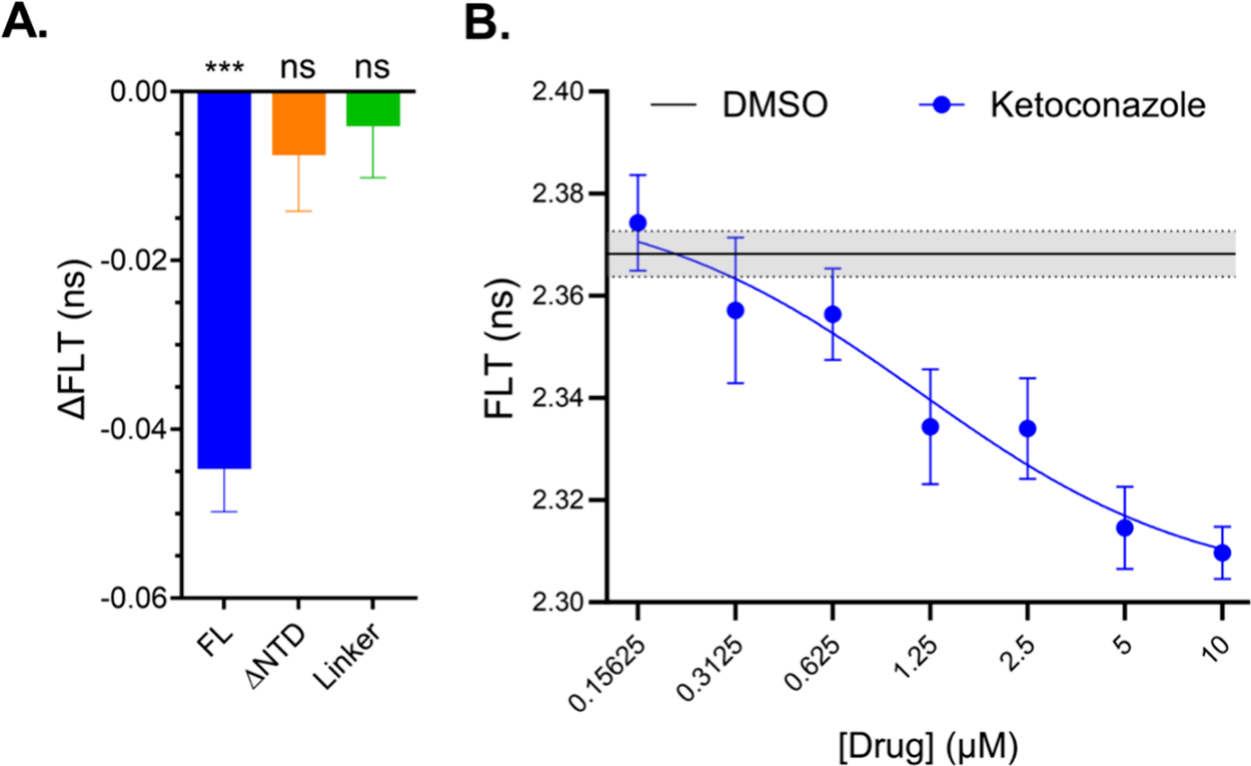
Ketoconazole modulates TDP-43 FLT in an NTD and dose-dependent manner. (A) ΔFLT for ketoconazole at 10 μM (screening condition) tested against FL, ΔNTD and linker biosensors. (B) FLT dose response for FL biosensor treated with ketoconazole 7-point titration resulted in EC_50_ = 1.2 μM. Statistics shown in (A) are one sample *t* tests to hypothetical mean of zero (DMSO baseline, ***p < 0.001). Data shown are mean ± SEM from *N* = 3–6 independent experiments.

To ensure that the decrease in FLT induced by ketoconazole is not associated with TDP-43 aggregation and puncta formation, we imaged TDP-43-mNg expressing HEK293T cells treated with 10 μM ketoconazole for 2 h (matching HTS conditions). Figure S12A-B shows that ketoconazole does not induce TDP-43 aggregation, consistent with its NTD-dependent effect. In contrast, when we tested erdafitinib — a FL TDP-43 hit that increased FRET in the ΔNTD biosensor — we observed widespread formation of puncta and mislocalization of TDP-43 (Figure S12C-D).

### Ketoconazole Increases High-Molecular Weight TDP-43 Cross-Linked Species

Next, we validated ketoconazole’s effect on TDP-43’s oligomerization via intact cell cross-linking of endogenous TDP-43 under the exact conditions used for FLT assays. To facilitate quantification of cross-linked oligomers, we subdivided the TDP-43 species into monomeric, 50–250 kDa cross-linked and >250 kDa cross-linked species. The 2-h ketoconazole treatments caused no change in total or monomeric TDP-43 levels (Figure S13A-B) but caused a significant increase in 50–250 kDa cross-linked species at 40 μM (Figure S13C), and a significant dose-dependent response in high molecular weight (>250 kDa) species (Figure S13D). Importantly, DMSO had no significant effect relative to untreated cells (Figure S14). Figure S13 shows levels of the three distinct TDP-43 populations normalized to the untreated cell control for each independent experiment. Since there was no significant change in total TDP-43 levels, we hypothesized that the increase in cross-linked species occurs with a concomitant decrease in the monomeric TDP-43 pool. Lane analysis was performed to determine the relative amounts of each population. Although not significant, there is a trending increase in the 50–250 kDa and >250 kDa TDP-43 species induced by 40 μM ketoconazole with concomitant reduction in the monomeric pool (Figure S15). Western blots used for Figure S13-15 are shown in Figure S16. Taken together, the lack of puncta in our live cell imaging and increase in HMW cross-linked TDP-43 species suggests ketoconazole modulates the formation of nuclear ribonucleoprotein (RNP) complexes, consistent in size to previously reported TDP-43 nuclear RNPs.^36^

### Ketoconazole Rescues Sorbitol-Induced Puncta and Cytoplasmic Mislocalization of TDP-43

We next explored whether ketoconazole could prevent the formation of TDP-43 puncta and mislocalization upon cell stress induction. Sorbitol-induced hyperosmotic stress has been widely used to elicit TDP-43 stress responses, and recent studies have shown that it disrupts TDP-43 NTD-interactions, leading to puncta formation and cytoplasmic mislocalization.^15,37,38^ Most sorbitol-induced hyperosmotic stress assays have used very high levels of insult (e.g., 0.4 M), but recent findings suggest lower concentrations are more effective at inducing mislocalization.^39^ We first optimized the sorbitol treatment conditions to establish the minimal perturbation that would result in significant changes in the formation of TDP-43 puncta and cytoplasmic mislocalization. Figure S17A-B shows that 0.1 M sorbitol is sufficient to induce puncta formation and mislocalization of TDP-43, and results in a similar FRET increase relative to vehicle for both FL and ΔNTD biosensors. This consistent FRET change suggests that sorbitol increases NTD-independent TDP-43 assemblies, providing an ideal paradigm to test whether stabilizing NTD-dependent interactions can block TDP-43 cytoplasmic mislocalization and aggregation.

Live cell imaging in Figure 4A shows that after 20 h of treatment, sorbitol causes widespread TDP-43 puncta and mislocalization, which is mitigated by 10 μM ketoconazole. Quantification of fluorescence imaging shows a statistically significant increase in TDP-43 cytoplasmic mislocalization due to sorbitol treatment relative to DMSO-only control, which is significantly reduced by ketoconazole (Figure 4B). In addition, Figure 4C shows that ketoconazole is able to rescue the sorbitol-induced increase in TDP-43 puncta to DMSO-only treatment levels. Figure S18 shows the treatment paradigm and time traces grouped by vehicle and sorbitol treatments.

**Figure 4:**
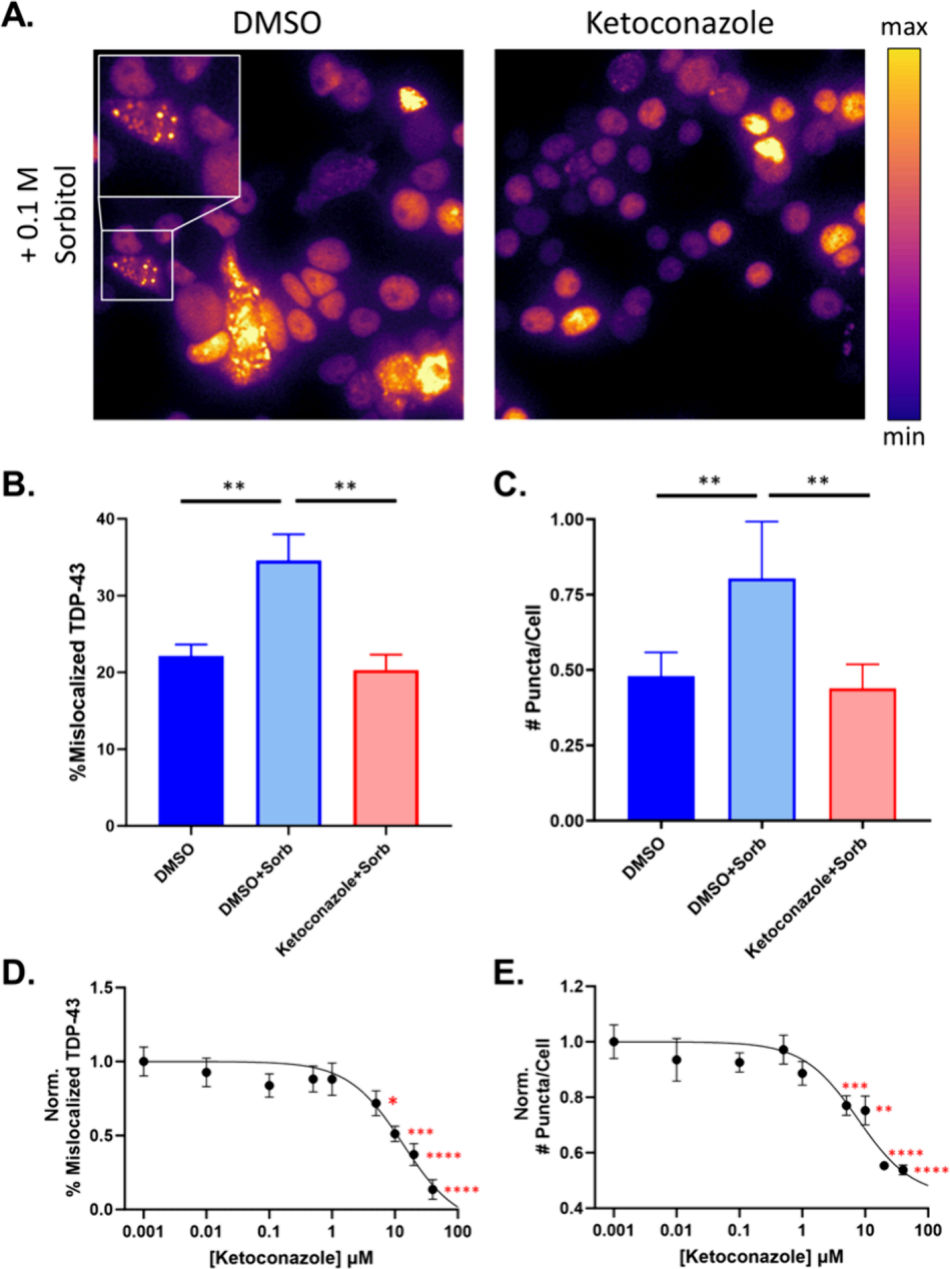
Ketoconazole rescues sorbitol-induced TDP-43 puncta formation and cytoplasmic mislocalization. (A) Fluorescence live cell imaging of HEK293T cells expressing FL TDP-43-mNg treated with 0.1 M sorbitol under DMSO or 10 μM ketoconazole treatments. Green fluorescence was mapped to a pseudocolor LUT. (B) End point quantification of puncta per cell. (C) End point quantification of TDP-43 cytoplasmic mislocalization. Statistics shown in (B–C) are two-way ANOVA multiple comparisons to DMSO-only control (solid blue) with Bonferroni correction (*p < 0.05, **p < 0.01). Figure S18 shows the full data set of puncta and mislocalization experiments. (D) Normalized mislocalized TDP-43 as a function of ketoconazole concentration (IC_50_= 13.42 μM). (E) Normalized number of puncta per cell as a function of ketoconazole concentration (IC_50_ = 7.65 μM). Statistics shown in (D-E) are ordinary one-way ANOVA multiple comparisons to DMSO-only controls with Bonferroni correction (*p < 0.05, **p < 0.01, *** *p* < 0.001, ****p < 0.0001). Data shown are mean ± SEM from *N* = 3 independent experiments.

Ketoconazole had a strong effect in both puncta and mislocalization assays; and thus, we tested whether dosing in the nanomolar to micromolar range had a titratable effect on these two phenotypes. Figure 4D-E shows that ketoconazole has a dose responsive effect on mislocalization (IC_50_ = 13.42 μM) and puncta formation (IC_50_ = 7.65 μM) under sorbitol treatment and is able to rescue mislocalization to a greater extent than puncta formation.

### Ketoconazole Rescues Loss of Neurite Outgrowth in TDP-43 Overexpression Model

Next, we tested ketoconazole’s ability to rescue TDP-43 induced neurite loss in a neuronal TDP-43 proteinopathy model in differentiated Neuro2a (N2a) cells stably overexpressing FL TDP-43-mNg. Neurite outgrowth has been recently used as a robust phenotypic assay that monitors ALS associated cellular dysfunction and for the discovery of novel ALS therapeutics.^6^ Stable FL TDP-43-mNg expression reduced neurite outgrowth by roughly 50% compared to naïve N2a cells (Figure 5A-B). Treatment of stable TDP-43-mNg expressing cells with 10 μM ketoconazole restored neurite outgrowth to 75% of naïve N2a cells, with DMSO-controls having no significant effect (Figure 5A-B).

**Figure 5:**
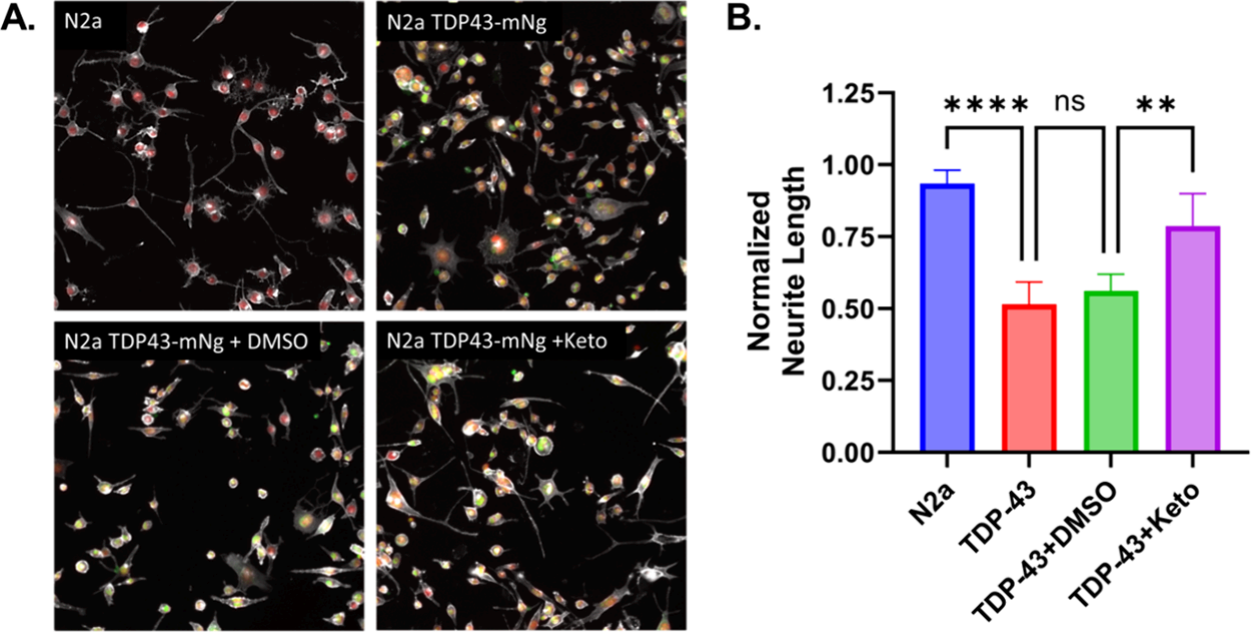
Neurite growth assay in retinoic acid differentiated normal and stable TDP-43-mNg expressing N2a cells. (A) Fixed cell fluorescence imaging of N2a and N2a TDP-43-mNg (untreated, DMSO and 10 μM ketoconazole) stained with phalloidin rhodamine (gray pseudocolor), Hoechst (red pseudocolor). mNeonGreen channel shown in green. (B) Normalized neurite length (total neurite length normalized to number of cells in each image) for each condition. Statistics shown are two-way ANOVA multiple comparisons to untreated TDP-43-mNg cells adjusted with Bonferroni correction. Data shown are mean ± SEM from *N* = 3 independent experiments with at least 6 images per condition for each run.

To rule out this phenotype being an artifact of tagging TDP-43 with a fluorophore, we conducted neurite length measurements of transiently transfected N2a cells with mNeonGreen, mCherry, TDP-43-mNg, TDP-43-mCh and TDP-43-mNg/TDP-43-mCh (at a 1:1 ratio with constant total TDP-43 DNA). Figure S19 shows no reduction in neurite length due to mNg or mCh expression relative to untransfected N2a cells. Furthermore, mNeonGreen and mCherry-tagged TDP-43 expressed separately or in combination caused a ∼ 20% reduction in neurite length relative to untransfected N2a cells (phenotype is less severe relative to stable cell line due to transient expression conditions).

### Ketoconazole Improves Motor Behavior in TDP-43 Overexpressing 

We then explored ketoconazole’s ability to rescue a TDP-43 induced pathological phenotype in a model of TDP-43 proteinopathy. The OW1601 strain developed by Koopman et al. expresses human TDP-43 (hTDP-43) pan-neuronally and exhibits motor deficits that are accompanied by formation of TDP-43 insoluble aggregates.^40,41^ Both control (OW1603) and hTDP-43 (OW1601) synchronized L1 stage animals were grown to L4 stage in DMSO or 10 μM ketoconazole. Motor deficits were monitored via a swimming assay which tracked body bends per 30 s (BB per 30s).

Figure 6 shows the hTDP-43 strain has a significantly reduced number of BB per 30s while swimming relative to controls. Ketoconazole treatment improves motor function in the hTDP-43 animals more than 2-fold relative to DMSO (∼16% to ∼ 40% of healthy controls). Importantly, ketoconazole does not enhance the motor function of control animals, suggesting a TDP-43-specific effect in the hTDP-43 strain. See supporting Movies S1–S6 for representative swimming assay recordings of all treatment conditions shown in Figure 6.

**Figure 6:**
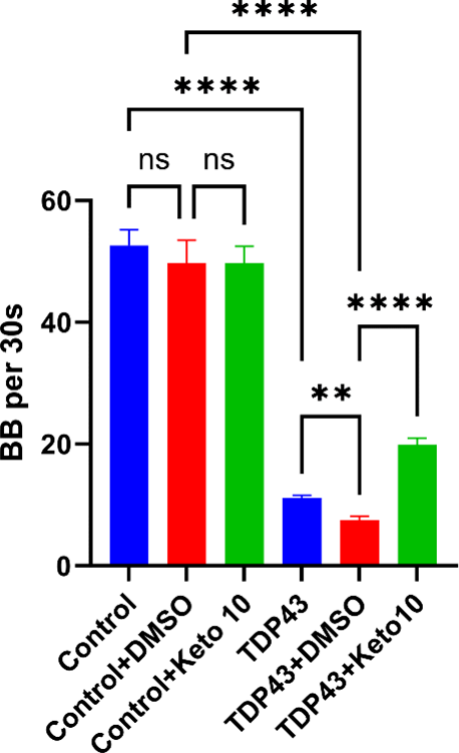
Ketoconazole improves motor dysfunction in human TDP-43 overexpressing OW1601 strain. BB per 30s for L4 stage control OW1603 strain and hTDP-43 strain OW1601 under DMSO or 10 μM ketoconazole treatment. Statistics shown are one-way ANOVA multiple comparisons with Bonferroni correction (**p < 0.01, ****p < 0.0001). Data shown are mean ± SEM from *N* = 3 batches containing at the minimum 10 animals.

### Ketoconazole Rescues TDP-43-Induced SREBP2 mRNA Deficiency

Next, we explored the potential MOA of ketoconazole. Ketoconazole is an antifungal and known binder and inhibitor of the human P450 cytochrome lanosterol 14-alpha demethylase (CYP51A1), which converts lanosterol to cholesterol in the last enzymatic step of the cholesterol biosynthesis pathway (see Figure 7A).^42^ Inhibition of CYP51A1 has been shown to upregulate the master regulator of the cholesterol biosynthesis pathway SREBP2 as a compensatory mechanism to restore homeostatic cholesterol levels.^42–46^

**Figure 7:**
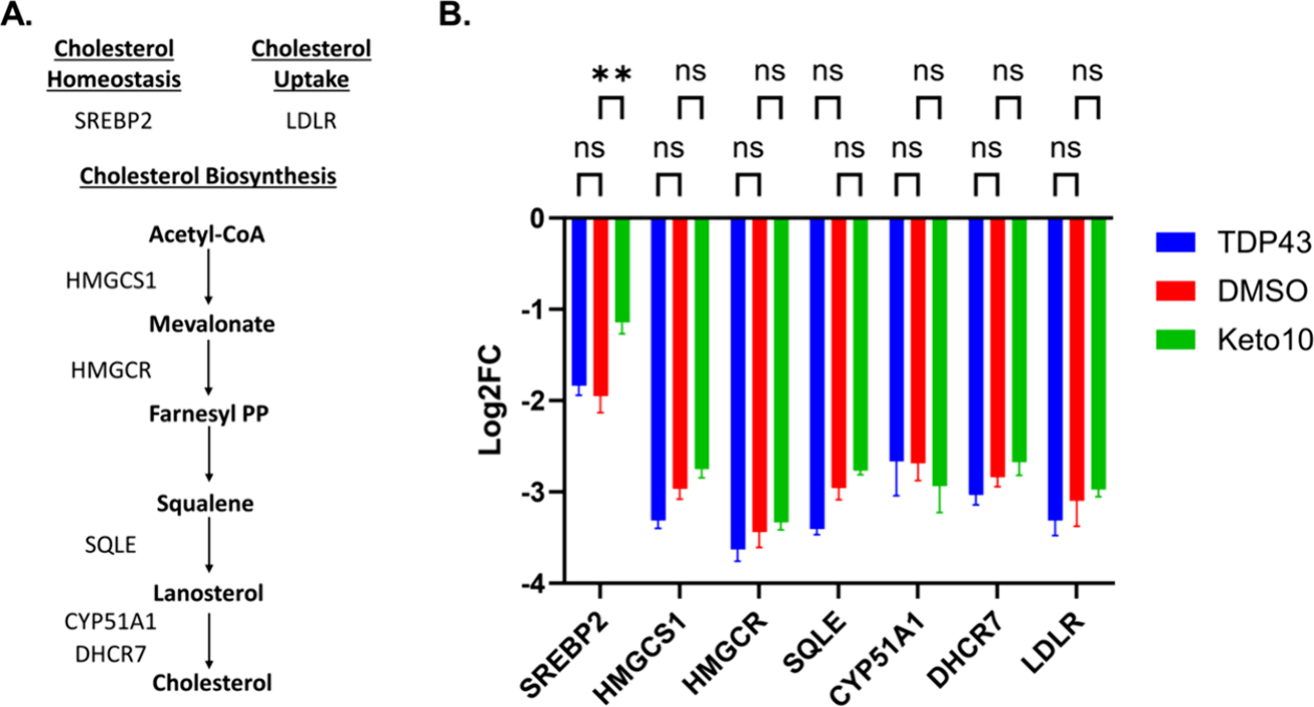
Ketoconazole partially restores SREBP2 mRNA under TDP-43 overexpression. (A) Summary of cholesterol biosynthesis pathway with annotated transcripts probed via RT-qPCR. (B) Log2FC of mRNA cholesterol biosynthesis genes under TDP-43 overexpression and DMSO/ketoconazole treatment probed via RT-qPCR. Log2FC values were calculated relative to nontransfected HEK293T cells and using GAPDH as a housekeeping gene. Statistics shown are one-way ANOVA multiple comparisons with Bonferroni correction against DMSO-treated TDP-43 overexpressing cells (red bar, **p < 0.01). Data shown are mean ± SEM from *N* = 3 independent experiments.

Recently, SREBP2 and the cholesterol biosynthesis pathway have been implicated in the pathogenesis of ALS and FTD.^47–49^ SREBP2 acts as a transcription factor when cleaved into N-terminal SREBP2 (nSREBP2) and regulates the expression of multiple enzymes in the cholesterol biosynthesis pathway via binding to sterol-regulated elements in DNA promoter regions.^47,48^ Inhibition of its activity can have detrimental effects on overall sterol homeostasis and myelination.^47,48^ This effect has been shown in HEK293T cells, spinal cord tissue of A315T mutant TDP-43 mice, mouse oligodendrocytes, and cerebrospinal fluid (CSF) samples of ALS patients.^47,48^

To explore this potential MOA, we mined De Abrew et al.’s publicly available transcriptomic data of four cancer cell lines (HepG2, MCF7, HepaRg and Ishikawa cells) treated with 34 different small molecules for 6 h, including ketoconazole.^50^ Indeed, at 10 μM dosing (matching the FLT-FRET experimental conditions) ketoconazole increased the expression of SREBP2, along with 14 out of 15 downstream SREBP2-regulated mRNAs that code for proteins in the isoprenoid and cholesterol biosynthesis pathway.^48^ Interestingly, TDP-43 is known to bind to and regulate 11 of these 15 proteins at the mRNA level (Figure S20A-B).^48^ The relatively small, but significant increase in SREBP2 in comparison to the SREBP2’s regulated genes suggest that modulating this hub has a greater overall effect and warrants further exploration in future studies. As a negative control, a similar analysis of amoxicillin, which was part of the Selleck library but not a hit in the FLT-FRET screens, does not cause any significant change relative to vehicle for these mRNAs (Figure S20A-B).

Next, using quantitative reverse transcription polymerase chain reaction (RT-qPCR) we examined whether the TDP-43 biosensor model recapitulates pathological SREBP2 downregulation and if ketoconazole is able rescue this deficit. TDP-43 overexpression caused a significant 2-fold reduction in SREBP2 levels relative to untransfected HEK293T cells (Figure 7 and Figure S21A for confirmation of TDP-43 overexpression). Ketoconazole significantly improved SREBP2 levels by 1-fold relative to DMSO-treated TDP-43 overexpressing cells, with DMSO showing no effect relative to untreated. We then expanded our inquiry to include the cholesterol pathway genes most affected by TDP-43 dysfunction (HMGCS1, HMGCR, SQLE, CYP51A1 (ketoconazole’s known target), DHCR7 and LDLR),^47,48^ whose role in the cholesterol biosynthesis pathway is shown in Figure 7A. TDP-43 overexpression caused a significant downregulation in all of these mRNAs with no significant rescue by ketoconazole relative to DMSO (Figure 7B), despite an overall trending increase (ketoconazole elevated mRNA levels of all transcripts relative to DMSO except CYP51A1). The selective effect of ketoconazole on SREBP2 mRNA but not its downstream genes could be due to an effect induced by TDP-43 overexpression, as TDP-43 regulates these targets at the mRNA level.

As a control, we also probed for autoregulation of TDP-43 mRNA. Overexpression of unlabeled TDP-43 induced a downregulation of endogenous TDP-43 (detected with 3′-UTR specific primers that do not detect mRNA transcribed from the TDP-43 plasmid), which is expected and consistent with TDP-43’s known autoregulatory mechanism (Figure S21B),^51^ with ketoconazole not showing an additional effect on TDP-43 levels.

### Inhibition of SCAP Blocks Ketoconazole-Induced High Molecular Weight TDP-43 Species

We have shown that ketoconazole is able increase SREBP2 mRNA levels and induce a dose-dependent formation of HMW (>250 kDa) TDP-43 assemblies, which match the reported molecular weights of nuclear TDP-43 RNPs.^36^ Since TDP-43 binds to multiple motifs in SREBP2 mRNA, we hypothesized that the increase in TDP-43 HMW cross-linked species may be mediated by SREBP2 activation into nSREBP2 (the cleaved nuclear form), as this species mediates the upregulation of SREBP2 mRNA. To further explore this MOA, we cotreated HEK293T cells with ketoconazole and betulin, a pharmacological inhibitor of SCAP (SREBP2 cleavage activating protein), the cholesterol sensitive enzyme that escorts SREBP2 to the Golgi apparatus for full-length SREBP2 cleavage into the transcription factor nSREBP2.^52,53^ We hypothesized that SCAP inhibition would counteract the ketoconazole-induced HMW TDP-43 cross-linked species by blocking the activation of SREBP2 into nSREBP2, as described in Figure S22A. As shown in Figure S22B-C, betulin treatment abolished the ΔFLT signal and >250 kDa cross-linked species induced by ketoconazole as measured by FLT and immunoblotting, suggesting that SREBP2 activation into nSREBP2 plays a role in the induction of HMW TDP-43 species. By itself, betulin increased FLT (decreased FRET) and lowered the HMW cross-linked TDP-43 species (Figure S22B), consistent with our proposed mechanism. See Figure S23-24 for Western blots used for quantification shown in Figure S22C. We ruled out cellular toxicity as a mediator of these observations via propidium iodide staining and flow cytometry, which shows no toxicity beyond DMSO levels by single or cotreatments (Figure S25).

### Other Hit Compounds of Interest

For the present study, we focused on NTD-dependent hits that met the counter-screening criteria (e.g., ketoconazole). Although ketoconazole was the only compound with no significant ΔNTD biosensor response — passing the stringent ΔNTD counter-screen filter — some compounds displayed a reduced ΔNTD biosensor response but under the conditions tested were not completely NTD-dependent. Two such hits, ginsenoside Rb1 and rifabutin, also have a known MOA related to the cholesterol biosynthesis pathway.^54,55^ Both ginsenoside Rb1 and rifabutin had a partial NTD-dependent ΔFLT effect (Figure S26) and were able to partially rescue TDP-43 mislocalization, puncta formation due to sorbitol (Figure S27) and SREBP2 levels due to TDP-43 overexpression (Figure S28, only ginsenoside Rb1 had a significant effect relative to DMSO). Another partial NTD-dependent compound, radotinib, is a C-abl kinase inhibitor — an MOA that is being pursued for ALS— and is currently being explored as a potential Parkinson’s disease therapeutic.^56,57^ These results suggest that broadening the counter-screen criterion to include partial NTD-dependent molecules may enrich for additional compounds with activity toward TDP-43.

As part of the hit criterion for this study, we only focused on compounds that reduced NTD-dependent FLT (increased FRET, indicating enhanced NTD-dependent interactions). Six hit compounds displayed an increase in FLT (reduced FRET), with three such compounds having an NTD-dependent response (Figure S11C). Indeed, the hit auranofin was previously identified in a screen targeting TDP-43 aggregation,^5^ suggesting that compounds like these may warrant further investigation as possible tool compounds that alter the distribution of toxic TDP-43 assemblies.

## Discussion

The screening platform developed here successfully identified a small molecule that modulates NTD-dependent TDP-43 interactions in live cells, providing a novel and precise tool for therapeutic discovery while supporting the therapeutic premise proposed by Oiwa et al..^15^ The FLT-FRET biosensors are compatible with HTS, offering a 16-fold improvement in precision over fluorescence intensity-based methods and enabling time-resolved, dynamic measurements of protein–protein interactions in live cells. This dynamic capability provides an advantage over conventional biochemical approaches, such as cross-linking and Western blotting, which rely on static, non-native conditions that can disrupt protein–protein interactions. Additionally, FLT provides advantages over other biophysical methods for monitoring TDP-43 interactions, such as BiFC and NanoBiT, due to its reversible signal, which allows for the observation of both assembly and disassembly processes, and its superior sensitivity compared to intensity-based techniques.

By screening in live cells, this platform is capable of identifying compounds that modulate TDP-43 interactions directly (by binding to TDP-43) or indirectly (by activating or inhibiting TDP-43-related pathways). Recent studies show that both direct and indirect targeting of TDP-43 can lead to novel therapeutics. For example, the NTD direct binder, nTRD22, ameliorates motor behavior deficits induced by TDP-43 overexpression in Drosophila;^58^ and ropinirole, an ALS drug currently in clinical trials, improves TDP-43 pathology in patient-derived iPSC motor neurons indirectly through modulation of the cholesterol biosynthesis pathway.^6^

A key strength of this platform is its ability to distinguish NTD-dependent effects through the counter-screening strategy that integrates FL and ΔNTD biosensors. Using this approach, ketoconazole emerged as a hit compound, demonstrating its ability to rescue multiple TDP-43-related pathological features. Specifically, ketoconazole, ameliorated TDP-43-dependent neurite outgrowth deficits in differentiated neurons, improved motor behavior in hTDP-43 , reduced sorbitol-induced TDP-43 puncta formation and cytoplasmic mislocalization, and improved the TDP-43-induced downregulation of SREBP2 (the master cholesterol biosynthesis regulator).

Although the direct connection between the cholesterol pathway and TDP-43 is not yet fully understood,^59^ our findings on ketoconazole implicate CYP51A1 inhibition as a factor influencing TDP-43 NTD-dependent oligomerization. Ketoconazole is an inhibitor of lanosterol 14-alpha demethylase (CYP51A1), known to reduce cholesterol levels, leading to the activation of SREBP2.^42–46^ We speculate that this activation is necessary for the induction of the HMW oligomeric TDP-43 species observed here, as pharmacological inhibition of SREBP2 activation via betulin counteracted ketoconazole’s effect. Future experiments quantifying lanosterol, cholesterol and nSREBP2 levels under ketoconazole and betulin treatment will be necessary to validate the proposed mechanism. In addition, genetic manipulation of CYP51A1 (via overexpression or silencing) and inhibition with different CYP51A1 inhibitors will be crucial to fully understand the connection between inhibition of this step in cholesterol biosynthesis and TDP-43 multimerization.

SREBP2 and its regulated genes contain multiple GU-rich TDP-43 binding sites,^48^ suggesting ketoconazole’s MOA on TDP-43 may be indirect by sequentially increasing SREBP2 transcription and sequestering nuclear TDP-43 into native functional oligomers. Indeed, Zhang et al. recently showed that multivalent GU-rich RNAs can sequester TDP-43 in the nucleus by inducing high molecular weight RNP complexes.^60^ This indirect MOA highlights the potential for targeting metabolic pathways (e.g., SCAP/SREBP2) to modulate TDP-43 activity and will be a focus in future investigations. Importantly, we did not rule out direct engagement of TDP-43 by ketoconazole as it was outside the scope of this project. Nevertheless, this remains a possibility and could be explored in future studies using established methods to monitor small molecule and TDP-43 interactions such as SPR or HSQC NMR.^58^

The modular design of this platform enables its application to other TDP-43 domains, broadening its utility. For example, more refined NTD disruptions, such as the 6M mutant (E14/17/21A, Q34A, R52/55A),^35^ could replace full domain deletions to specifically inhibit NTD-mediated oligomerization. Additionally, modifications to the nuclear localization/export sequences, RRMs, or CTD could bias the ensemble of TDP-43 states, facilitating domain-specific screening under diverse conditions. For example, in a recent study by Yang et al., they designed an aggregation-resistant CTD phosphomimetic mutant (S333D/S342D) to identify small molecules that stabilize TDP-43’s native state in a cell-free platform.^8^ The versatility of our counter-screening strategy allows exploration of domain specific TDP-43 aggregation dynamics, FTD/ALS-related mutations, and the impact of ALS/FTD-risk factors like neuroinflammation or environmental toxins on TDP-43 assemblies.^61–66^

In summary, this screening platform offers unparalleled sensitivity and dynamic capabilities for identifying modulators of NTD-dependent TDP-43 interactions. The counter-screening strategy reliably differentiates domain-specific effects and highlights promising compounds, such as ketoconazole, that rescue multiple TDP-43 insults through pathways like SREBP2-mediated cholesterol regulation. Future studies should leverage this platform to expand the understanding of TDP-43 dynamics, domain-specific interactions, and their implications for ALS/FTD pathogenesis.

## Materials and Methods

### Cell Culture

HEK293T cells (ATCC) were maintained in full Gibco DMEM media (10% FBS, 1% Pen/Strep, 1% GlutaMax) at 37 °C, 5% CO2, and humidity control. All transfections were carried out using Thermo Fisher Lipofectamine 3000 at a DNA:P3000:L3000 ratio of 1:2:3 (μg DNA: μL P3000: μL L3000), with 2.5 μg DNA per 1e6 seeded cells.

N2a cells (ATCC) were maintained in full EMEM media (10% FBS, 1% Pen/Strep, 1% GlutaMax, 1% NEAAs) and differentiated in 0.5% FBS with 10 μM retinoic acid. Stable FL TDP-43-mNg expressing N2a cells were generated by transfection with Lipofectamine 3000, followed by selection with Zeocin and cell sorting using flow cytometry.

### Western Blotting

Cells were harvested using trypsin (Gibco) and washed in PBS (Sigma-Aldrich) once prior to lysis with (ultrapure water, TNE buffer, 1% SDS, 0.5% DOC, 0.5% NP-40, phosphatase and kinase inhibitors). Samples were incubated on ice for 10 min, sonicated at 50% amplitude for 3x 3s pulses, boiled at 95 °C for 10 min and spun down at 21,300g for 10 min. Using the supernatant, bicinchoninic acid assays (BCA, Thermo Scientific) were conducted to quantify total protein content and samples prepared in 1x Laemmli buffer (BioRad) with beta-mercaptoethanol.

SDS-PAGE gels were run at 120 V using 12% BioRad gels with 10–20 μg of protein loaded in each well. Gels were transferred into nitrocellulose membranes using the BioRad TurboTransfer protocol and stained for total protein using Ponceau S. Finally, blots were probed with TDP-43 (Proteintech 10782–2-AP), mNeonGreen (Cell Signaling Technology E6M3D) and mCherry (Cell Signaling Technology E5D8F) primary antibodies at 1:2000 dilution overnight at 4 °C and detected using HRP-conjugated secondary antibodies at 1:10000 dilution imaged with SuperSignal West Pico PLUS chemiluminescent substrate (Thermo Scientific).

For cross-linking TDP-43 Western blots (Figure S16, Figure S23 and Figure S24), the full blot was imaged first and then cut above ∼ 50 kDa and reimaged to obtain better resolution (and less bleed-through from monomer bands) for cross-linked species. Monomeric levels were quantified from the full blot, whereas 50–250 kDa and >250 kDa species were quantified from the cropped blot. Lane analysis for relative amounts of species was performed on the full blot.

### Intact Cell Cross-Linking

DSG cross-linking shown in Figures S5–8 was conducted by incubating 1e6 PBS-washed cells resuspended in 500 μL with 200 μM DSG (Sigma-Aldrich) for 30 min under gentle rotation. Next, the cross-linking reaction was quenched with 20 mM Tris (pH 7) for 15 min at room temperature. After quenching, cells were spun down and processed for Western blotting as described above. For cross-linking experiments involving ketoconazole or betulin treatment (Figure S16, Figure S23 and Figure 24), cells were incubated in PBS under constant rotation for 2 h, spun down and resuspended in PBS containing 125 μM DSG following all steps described above.

### Cell Preparation and FLT-FRET Measurements

For all FLT-FRET experiments, cells were transfected with 0.5 μg/1e6 cells TDP-43-mNg (donor-only), 0.5 μg/1e6 cells TDP-43-mNg + 0.5 μg/1e6 cells TDP-43-mCh (1:1 FL), 0.5 μg/1e6 cells TDP-mNg + 0.5 μg/1e6 cells TDP-43-mCh ΔNTD (1:1 ΔNTD) or 0.5 μg/1e6 cells mCh-Linker-mNg complemented with empty vector plasmid to reach 2.5 μg DNA/1e6 cells. After 24 h of transfection, cells were lifted using trypsin, washed in PBS twice and resuspended at a final concentration of 1e6 cells/mL. 50 μL of cells were added to black-bottom 384-well plates where FLT measurements were collected.

FLT measurements were made in a fluorescence lifetime plate reader (Fluorescence Innovation, USA) equipped with a 473 nm laser, 488 dichroic, and dual channel detection at 517/20 (primary) and 535/6 (secondary).^25^ FLT for each well is calculated by fitting a single exponential decay function to the time-resolved fluorescence waveforms as described previously.^25^ FRET efficiency was then calculated using eq 1.

$$FRET=1-\frac{\tau_{DA}}{\tau_{D}}$$ 1

### Cell Preparation and FDA-Approved Selleck Library FLT-FRET Screens and Dose Responses

Cells were transfected with full-length and linker-only biosensors in 15 cm plates using the same DNA concentrations listed previously. After 24 h of transfection, cells were lifted, washed in PBS twice, passed through a cell strainer, and resuspended to 1e6 cells/mL. The Selleck library used consists of 2684 FDA-approved small molecules predispensed across three separate 1536-well plates, which include empty and DMSO wells as negative controls. After equilibrating the plates to room temperature, 5 μL of cells were dispensed in each well using a Thermo Fisher Multidrop Combi for a final drug concentration of 10 μM. Plates were incubated at room temperature for 2 h prior to FLT-FRET measurements. TDP-43 and linker unique hits were determined by finding ±3SD hits that appeared in each independent screen, flagging interfering compounds with a spectral similarity filter as described previously.^25^ TDP-43 unique hits were defined as hits from the TDP-43 screen that did not show up as hits in the linker screen.

TDP-43 reproducible and unique hits were then tested by conducting FLT-FRET measurements at increasing concentrations (∼156 nM to 10 μM). The exact same steps and incubation times were followed for dose responses, with the only difference being that these experiments were conducted in 384-well plates predispensed with drugs (see chemical vendors in Table S1) using a PerkinElmer Flexdrop liquid dispenser.

### TDP-43 Puncta Formation and Mislocalization Imaging and Analysis

HEK293T cells were seeded at 0.16e6 cells per well in 24-well plates. After 24 h of incubation, each well was transfected with 0.08 μg TDP-43-Ng (supplemented with 0.32 μg empty vector for a total of 0.4 ug DNA per well, 2.5 μg DNA/1e6 cells). After 24 h of incubation, cells were pretreated with 0.2% DMSO control and 10 μM ketoconazole, ginsenoside Rb1 and rifabutin for 2 h. After 1.5 h, Hoechst stain was added at a 1:50k dilution and incubated for 30 min. Images shown in Figure S3 were collected at this stage. Next, ultrapure water control or 0.1 M sorbitol were added to wells. Plates were imaged for nuclear, TDP-43-mNg and brightfield channels continuously under environmental control (37 °C, 5% CO2, 15% O2 and humidity control) every hour for 20 h since addition of drug in a Molecular Devices Pico ImageXpress microscope.

Images were analyzed using MetaXpress High Content Image Analysis software with two custom analysis workflows that quantify number of puncta per cell and cytoplasmic mislocalization based on intensity thresholding and nuclear masking using the Hoechst channel.

### Neurite Length Assay

Neuro2a (N2a, ATCC) cells were plated at a density of 70K cells/well in a 24-well plate. After 6 h, 10 μM retinoic acid differentiation media was added to the wells. After 24 h of incubation, 10 μM ketoconazole or DMSO was added for an additional 24 h. Wells were washed in PBS, fixed in 4% paraformaldehyde, permeabilized with 0.5% Triton-X and stained with Hoechst and phalloidin rhodamine (Cytoskeleton, Inc.) following the manufacturers protocol. Images collected with a Molecular Devices Pico ImageXpress microscope were analyzed in MetaXpress using the nuclear and actin stain channels.

For transient transfection neurite length experiment shown in Figure S19, N2a cells were plated in a 24-well plate at a density of 60K cells/well and incubated for 24 h. Next, cells were transfected with mNg, mCh, TDP-43-mNg and TDP-43-mCh at a ratio of 2.5 μg DNA/1e6 cells, incubated for 8 h, at which point the media was switched to differentiation media containing 10 μM retinoic acid. Cells were incubated for 48 h, stained using phalloidin rhodamine and Hoechst, and imaged.

###  Motor Behavior Analysis via Swimming Assay

Control (OW1603, dvIs15 [unc-54­(vector) + mtl-2::GFP]) and human TDP-43 overexpressing (OW1601, dvIs62 [snb-1p::hTDP-43/3′ long UTR + mtl-2p::GFP] X) strains were obtained from Ellen A. A. Nollen from the University of Groningen and long-term stored at the *Caenorhabditis* Genomics Center (CGC) at the University of Minnesota. Animals were grown on nematode growth medium (NGM) plates at 21 °C. Drug plates were prepared using NGM agar supplemented with 0.2% DMSO or 10 μM ketoconazole in a 6 cm plate. Solidified plates were seeded with OP50 in 0.2% DMSO or 10 μM ketoconazole.

Animals were synchronized at L1 stage following established protocols.^67^ Hatched L1 larvae were distributed onto NGM agar drug plates (∼10 animals per well in triplicate wells per each condition). Worms were incubated at room temperature for 3 days. Worms were collected, washed in M9 buffer and plated in 12-well NGM agar plates containing sufficient M9 buffer for monitoring swimming behavior. Animals were allowed to equilibrate for 1 min before recording 30 s videos. The video recordings were processed and analyzed to quantify BB per 30s using the wrMTrck plugin in ImageJ.

### Mining of Ketoconazole Transcriptomics Data

Differences in expression of SREBP2 and its regulated genes between vehicle and ketoconazole or amoxicillin in HepG2, MCF7, HepaRg and Ishikawa cells were calculated using the data deposited by De Abrew et al. on NCBI’s Gene Expression Omnibus (GSE69851).^50^ The integrated GEO2R analysis suite was utilized to calculate log2FC expression changes of SREBP2-regulated genes in the cholesterol biosynthesis pathway.

### Real-Time Quantitative PCR of Endogenous TDP-43 and SREBP2

HEK293T cells were seeded at 0.8e6 cells per well in 6-well plates and incubated for 24 h. After incubation, cells were transfected with 2 μg of unlabeled full-length TDP-43 (2.5 μg DNA/1e6 cells). Two hours after transfection, cells were treated with 10 μM ketoconazole, ginsenoside Rb1 and rifabutin (as well as 0.2% matching DMSO control) and incubated for an additional 24 h.

Total RNA for each condition was isolated using Qiagen RNAeasy extraction protocol. After quality validation via 280/260 measurements, total RNA was reversed transcribed using Thermo Fisher High-Capacity RNA-to-cDNA kit. PCR reactions probing for endogenous GAPDH (housekeeping), TDP-43, SREBP2 and its regulated genes were prepared following the Thermo Fisher SYBR Green qPCR protocol and using the following specific primers (IDT) for endogenous mRNA (designed using NCBI’s primer designing tool or from Egawa et al.^47^):

GAPDH

Forward: 5′-AATGGGCAGCCGTTAGGAAA-3′
Reverse: 5′-GCGCCCAATACGACCAAATC-3′

TDP-43:

Forward: 5′-GAGAAAAGGAGAGAGCGCGT-3′
Reverse: 5′- GGGGTAGGGGGAGTACAAGT −3′

SREBP2:

Forward: 5′-TGTGTCCTCACCTTCCTGTGCCT-3′
Reverse: 5′-TCCAGTCAAACCAGCCCCCAGA-3′

HMGCS1

Forward: 5′-CCCCAGTGTGGTAAAATTGG-3′
Reverse: 5′-TGGCCTGGACTTAACATTCC-3′

HMGCR

Forward: 5′-GGACCCCTTTGCTTAGATGAAA-3′
Reverse: 5′-CCACCAAGACCTATTGCTCTG-3′

SQLE

Forward: 5′-GCTGCCTGTACATCAACATC-3′
Reverse: 5′-GACCAAGGTCTTTGAGAACAT-3′

DHCR7

Forward: 5′-ACTTTAGCCGGTTGAGAAGGA-3′
Reverse: 5′-CCCTTGAGATGCGGTTCTGT-3′

CYP51A1

Forward: 5′-CTCGTTCCGTCGATTGGGAG-3′
Reverse: 5′-TAGACCAGGCTGAGGGTGAA-3′

LDLR

Forward: 5′-GACGTGGCGTGAACATCTG-3′
Reverse: 5′-CTGGCAGGCAATGCTTTGG-3′

RT-qPCR reactions were conducted using a Roche LightCycler 96 plate reader and analyzed using Roche’s PCR analysis software. Expression was quantified using the 2^–ΔΔCt^ method relative to untreated/untransfected HEK293T cells.^68^

### Flow Cytometry

HEK293T cells were treated with DMSO (negative control), 10 μg/mL puromycin (positive control), 40 μM ketoconazole, 40 μM betulin and 40 μM ketoconazole/betulin for 2 h. Next, cells were stained using propidium iodide (Thermo Fisher) in PBS following the manufacturers protocol. Cells were flowed using a BD Accuri C6 flow cytometer until at least 25K events per condition were collected. Histograms of FL2 (585/40 nm) and median FL2 were generated and calculated using the BD Accuri C6 analysis software.

### Statistical Analysis

Multiple comparisons were conducted via ordinary one-way or two-way ANOVAs with posthoc Bonferroni corrections for multiple comparisons. Single comparisons were conducted via one sample *t* tests or unpaired two-tailed *t* tests. All statistical analysis was done in GraphPad Prism 9.

## Supplementary Material















## Data Availability

Fluorescence lifetime analysis was conducted using a single-exponential fitting script written in MATLAB. All data needed to evaluate the conclusions are present in the paper and/or the Supporting Information. The data sets or scripts generated in this study are available from the corresponding author on reasonable request.

## Notes

Table S1 (hit compound summary) and Figures S1–S28 (additional FRET, fluorescence imaging, immunoblotting, neurite outgrowth, transcriptomics, RT-qPCR, and flow cytometry experimental data) (PDF)
Representative swimming assay recording of untreated control (OW1603) (MOV)
Representative swimming assay recording of DMSO-treated control (OW1603) (MOV)
Representative swimming assay recording of 10 μM ketoconazole-treated control (OW1603) (MOV)
Representative swimming assay recording of untreated TDP-43 (OW1601) (MOV)
Representative swimming assay recording of DMSO-treated TDP-43 (OW1601) (MOV)
Representative swimming assay recording of 10 μM ketoconazole-treated TDP-43 (OW1601) (MOV)
