# Supplementary material for: Fluorescence Lifetime-Based FRET Biosensors for Monitoring N Terminal Domain-Dependent Interactions of TDP-43 in Living Cells: A Novel Approach for ALS and FTD Drug Discovery
Source: ACS Chem Neurosci. 2025 Jun 10;16(13):2450–62. doi: 10.1021/acschemneuro.5c00266 (PMC12232324; doi:10.1021/acschemneuro.5c00266)
Supplement: Supplementary file 1 [file cn5c00266_si_001.pdf]

Title

Fluorescence lifetime-based FRET biosensors for monitoring NTD-dependent interactions of TDP-43 in living cells: A novel approach for ALS and FTD drug discovery

Authors

Noah Nathan Kochen[1†], Marguerite Murray[1], Sophia Zafari[1], Nagamani Vunnam[1], Elly E. Liao[1], Lihsia Chen[2], Anthony R. Braun[1*], and Jonathan N. Sachs[1*]

[1]Department of Biomedical Engineering, University of Minnesota, Minneapolis, MN 55455.

[2]Department of Genetics, Cell Biology and Development, University of Minnesota, Minneapolis, MN 55455.

[†]First author; [*]co-corresponding author

Corresponding Authors: brau0123@umn.edu and jnsachs@umn.edu

This PDF file includes:

- Table S1
- Figs. S1 to S28
- Legends for Movies S1 to S6

Other Supplementary Materials for this manuscript include the following:

- Movies S1 to S6

	Drug Name	Delta Lifetime Z-Score	Standard Error	Selleck-Listed Target	Vendor
1	Danthron	-11.621	3.951	N/A	Sigma-Aldrich
2	Rifapentine	-6.702	0.192	DNA/RNA synthesis	Selleck
3	Radotinib	-5.867	1.165	BCR-Abl	Selleck
4	Sennoside A	-5.281	0.577	MAO	Selleck
5	Erdafitinib (JNJ-42756493)	-5.265	0.648	FGFR	Cayman Chemical
6	Rifamycin sodium salt	-5.004	0.991	Others	USP
7	Proanthocyanidins	-4.947	0.708	Others	Selleck
8	Dithranol	-4.412	1.605	Others	Oakwood Chemical
9	Levothyroxine sodium	-4.222	1.186	TR-alpha/beta	Sigma-Aldrich
10	Evans Blue	-3.480	1.029	GluR	Sigma-Aldrich
11	Nystatin (Fungicidin)	-3.416	0.648	Anti-infection	Cayman Chemical
12	Diacerein	-3.408	1.417	IL eceptor	Cayman Chemical
13	Ketoconazole	-3.308	0.534	P450	CHEM-IMPEX
14	Rifabutin	-3.120	0.062	Anti-infection	CHEM-IMPEX
15	Oxytetracycline Dihydrate	-3.024	0.234	Anti-infection	Selleck
16	Ruboxistaurin (LY333531 HCl)	-2.905	1.949	PKC	Chem Cruz
17	Ginsenoside Rb1	-2.747	0.275	Others	Selleck
18	Auranofin	3.721	2.003	Others	Selleck
19	Clindamycin palmitate HCl	3.856	0.619	Others	Selleck
20	Trapidil	4.298	0.557	PDGFR	Selleck
21	Omeprazole Sodium	4.450	0.215	Proton pump	Selleck
22	Allopregnanolone	6.526	3.088	GABA receptor	Spectrum Chemical MFG
23	Pomalidomide	8.543	2.405	TNFalpha	Cayman Chemical

Table S1. Summary of FDA-approved Selleck library TDP-43 unique hits. For each hit, the ΔFLT Z-score, standard error, Selleck-reported target and vendor for follow-up experiments are shown.

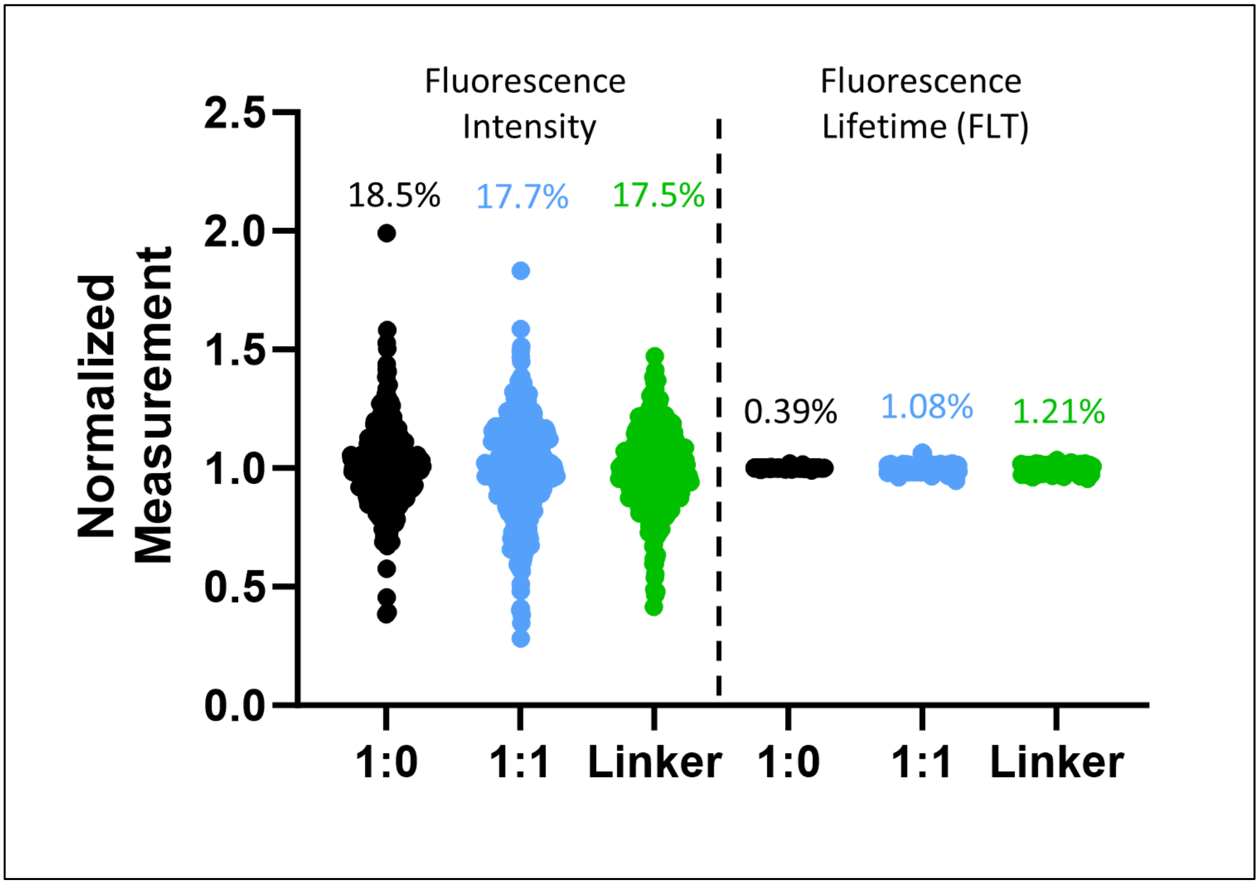

Fig. S1. Fluorescence lifetime is 16-fold more sensitive than fluorescence intensity. Normalized fluorescence intensity and fluorescence lifetime for 473nm excitation channel of TDP-43-mNeonGreen (1:0), FL TDP-43-mNeonGreen + FL TDP-43-mCherry (1:1) and mNeonGreen-Linker-mCherry (Linker) constructs used for monitoring TDP-43 FLT-FRET in live HEK293T cells. Percentages indicate % coefficient of variation (%CV) of 384 individual wells of a 1536-well plate.

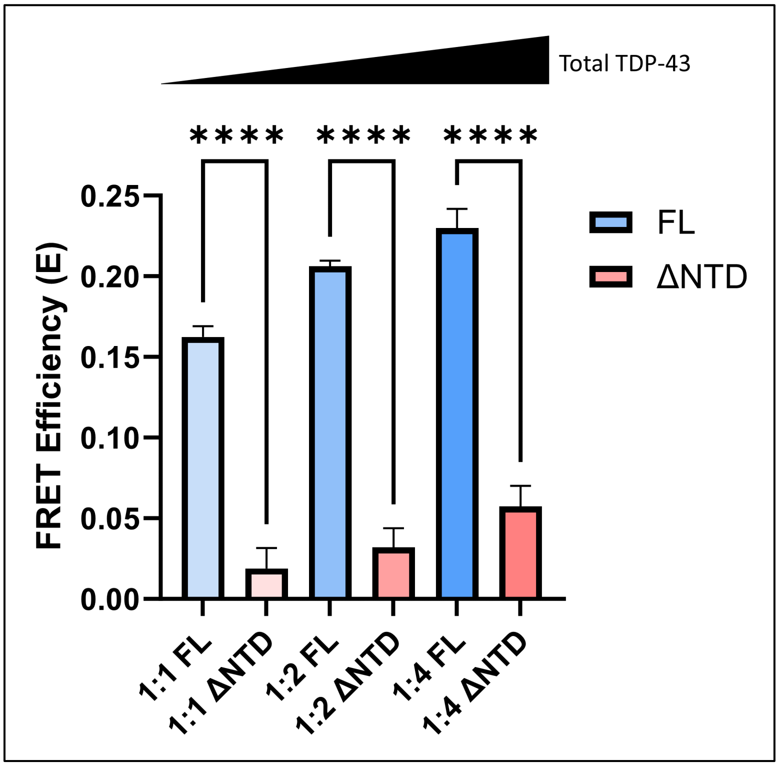

Fig. S2. Non-NTD dependent FRET increases with higher levels of TDP-43 biosensor expression. FRET efficiencies of FL and ΔNTD TDP-43 biosensors at different donor:acceptor ratios (1:1, 1:2, 1:4) and increasing total mass amount of biosensor plasmid transfected (0.8, 1.2, 1.6 µg). Statistics shown are one-way ANOVA multiple comparisons with Bonferroni correction (**** $p < 0.0001$). Data shown are mean ± SEM from N=3 independent experiments. First two bars are data shown in Fig. 1B for reference.

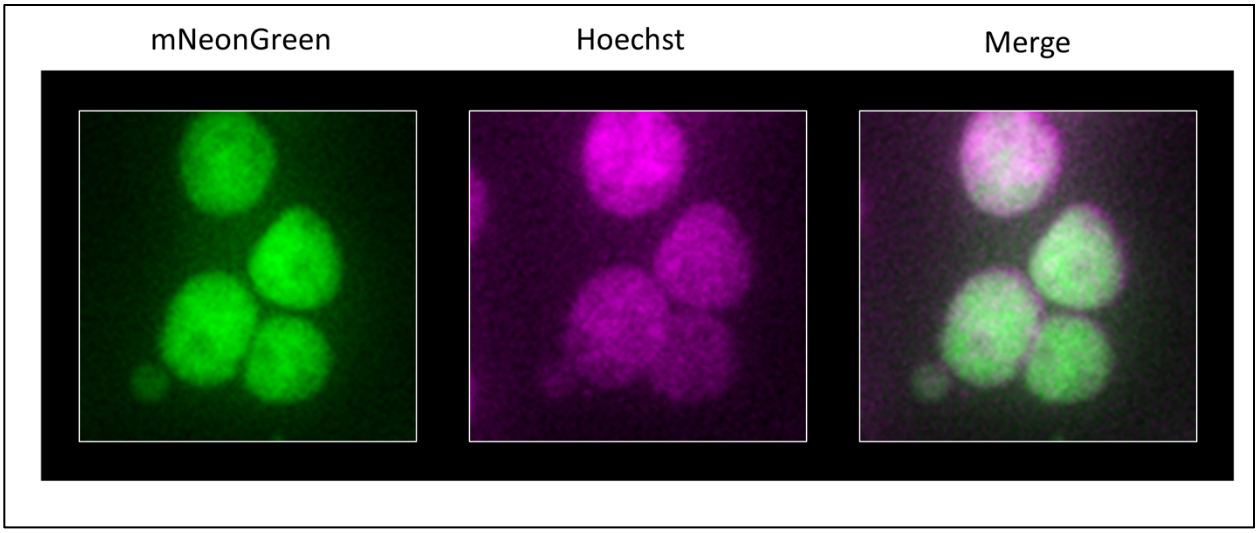

Fig. S3. Full-length TDP-43 biosensor subcellular localization. Fluorescence microscopy images of FL TDP-43-mNeonGreen expressing HEK293T cells transiently transfected for 24 hours. Hoechst nuclear stain is shown as magenta pseudo-color for better contrast.

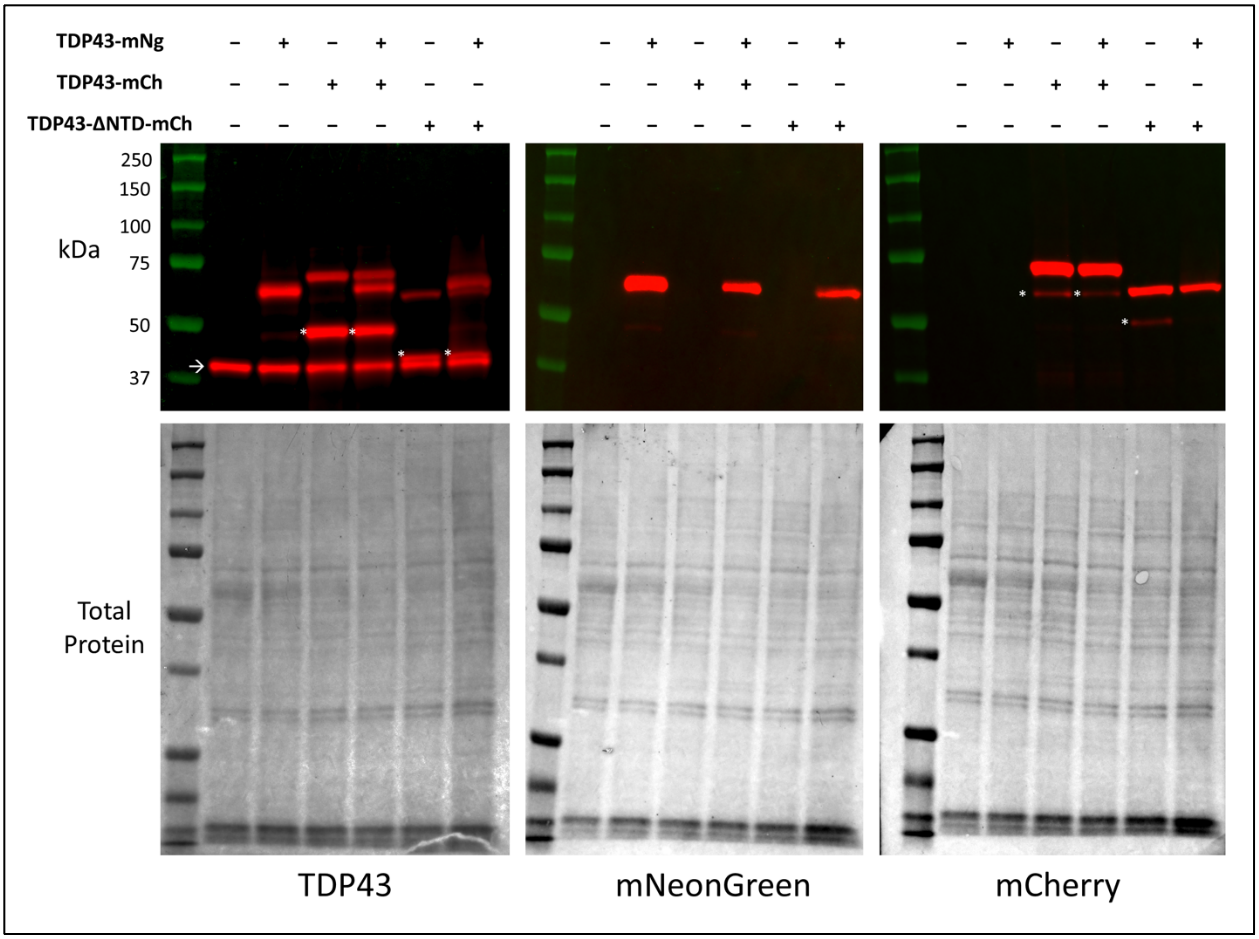

Fig. S4. Expression of mNeonGreen and mCherry FL and ΔNTD TDP-43 biosensors in HEK293T cells. Biosensor expression monitored via western blots probing for TDP-43, mNeonGreen and mCherry. Endogenous monomeric TDP-43 is marked with a white arrow (→). Both FL and ΔNTD TDP43-mCh constructs (Lanes 3 and 5 respectively) show a prominent truncation band that is TDP-43 antibody reactive and larger than endogenous TDP-43. mCherry has a known cleavage site that leads to truncation of the fluorophore (indicated with *) (*1*). In our FLT-FRET system we only monitor the donor FLT so the presence of soluble/truncate mCherry acceptor does not interfere with the FLT-FRET measurements. Loss of NTD-NTD interactions have been shown to increase TDP-43 turnover (*2*), which explains the reduced expression of ΔNTD biosensor relative to FL.

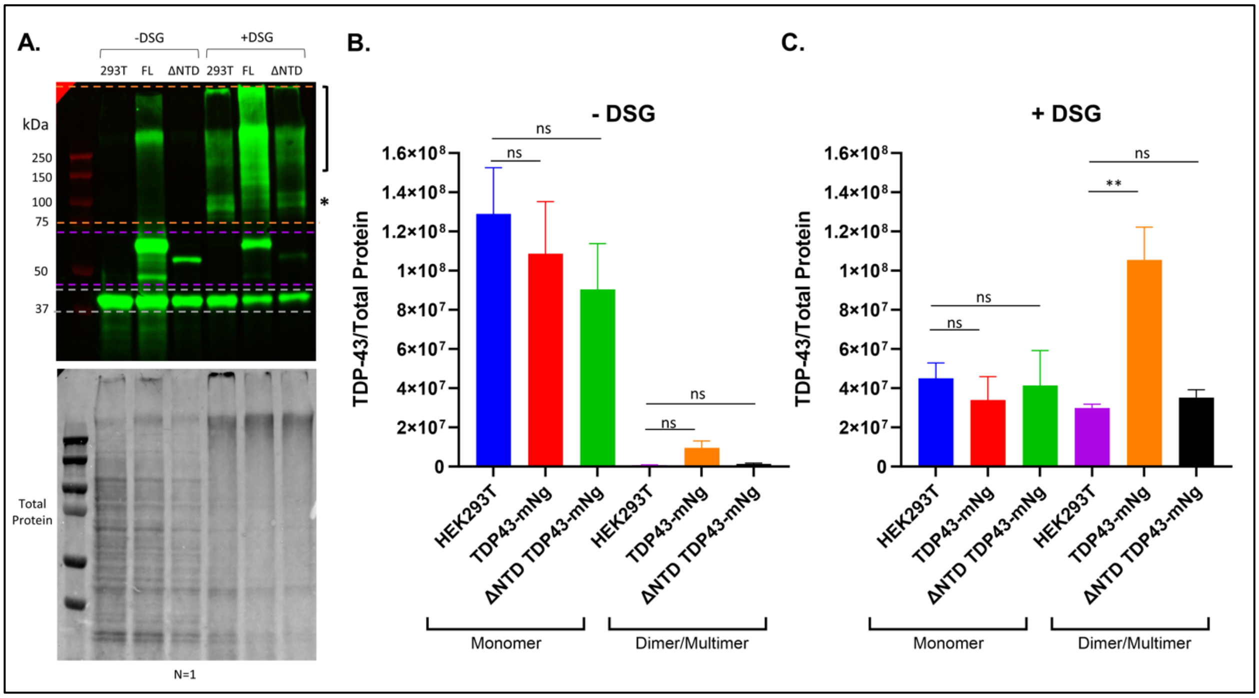

Fig. S5. Full-length mNeonGreen-tagged biosensor expression correlates with higher TDP-43 dimer/multimer levels. (A) Western blot of HEK293T cells expressing FL and ΔNTD TDP-43 donor biosensor with and without DSG crosslinking. Total protein was stained using Ponceau S. Regions delimited by grey, purple and orange dashed lines indicate endogenous monomer, biosensor monomer and cross-linked (dimer/multimer) TDP-43 species, respectively. Square brackets indicate multimeric species (>100 kDa), asterisk (*) indicates putative dimeric bands (neighboring 100 kDa). **(B)** Quantification of TDP-43 monomer and dimer/multimer levels in un-crosslinked samples. **(C)** Quantification of TDP-43 monomer and dimer/multimer levels in crosslinked samples. The two additional western blots used for quantification in (B-C) are shown in Fig. S6. Statistics shown are one-way ANOVA multiple comparisons with Bonferroni correction (**$p < 0.01$). Data shown are mean ± SEM from N=3 independent experiments.

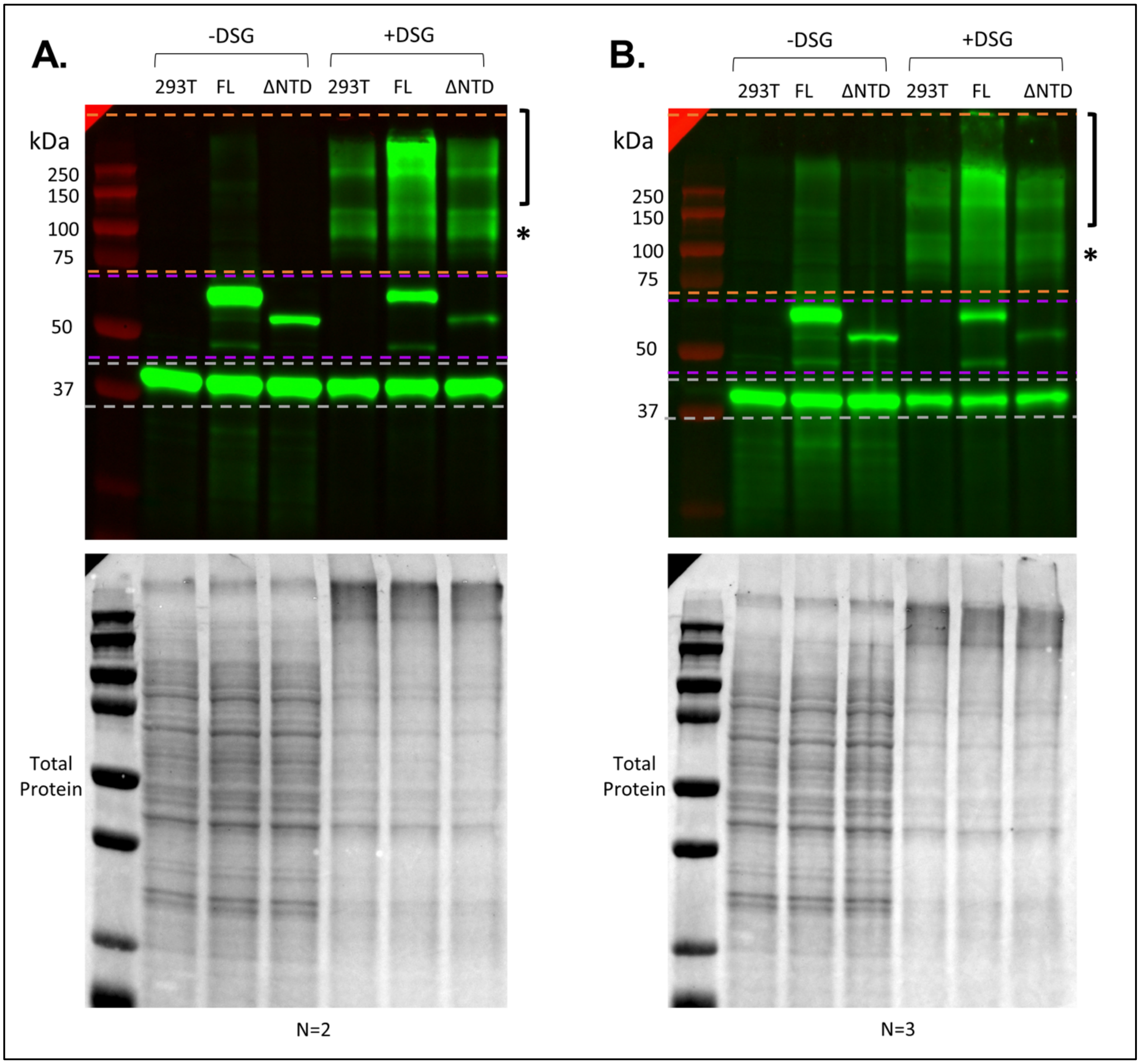

Fig. S6. Western blots of un-crosslinked and crosslinked HEK293T cells expressing FL and ΔNTD mNeonGreen biosensors. (A-B) Each blot shows an independent transfection, harvest and western blotting for TDP-43. Regions delimited by grey, purple and orange dashed lines indicate endogenous monomer, biosensor monomer and cross-linked (dimer/multimer) TDP-43 species, respectively. Square brackets indicate multimeric species (>100 kDa), asterisk (*) indicates putative dimeric bands (neighboring 100 kDa).

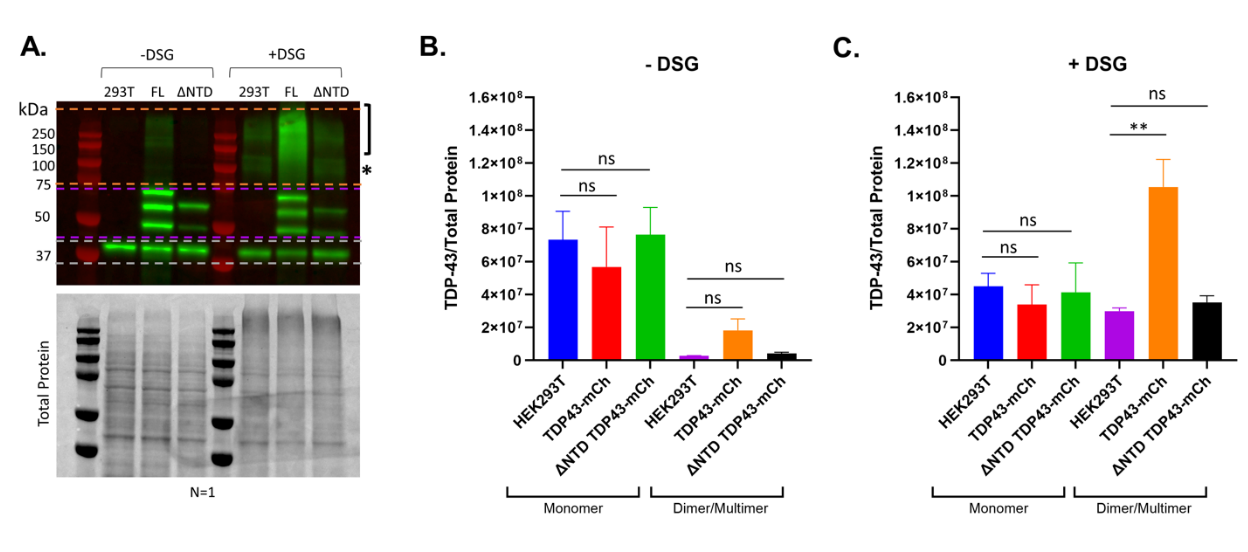

Fig. S7. Full-length mCherry-tagged biosensor expression correlates with higher TDP-43 dimer/multimer levels. (A) Western blot of HEK293T cells expressing FL and ΔNTD TDP-43 donor biosensor with and without DSG crosslinking. Total protein was stained using Ponceau S. Regions delimited by grey, purple and orange dashed lines indicate endogenous monomer, biosensor monomer and cross-linked (dimer/multimer) TDP-43 species, respectively. Square brackets indicate multimeric species (>100 kDa), asterisk (*) indicates putative dimeric bands (neighboring 100 kDa). **(B)** Quantification of TDP-43 monomer and dimer/multimer levels in un-crosslinked samples. **(C)** Quantification of TDP-43 monomer and dimer/multimer levels in crosslinked samples. The two additional western blots used for quantification in (B-C) are shown in Fig. S8. Statistics shown are one-way ANOVA multiple comparisons with Bonferroni correction (**$p < 0.01$). Data shown are mean ± SEM from N=3 independent experiments.

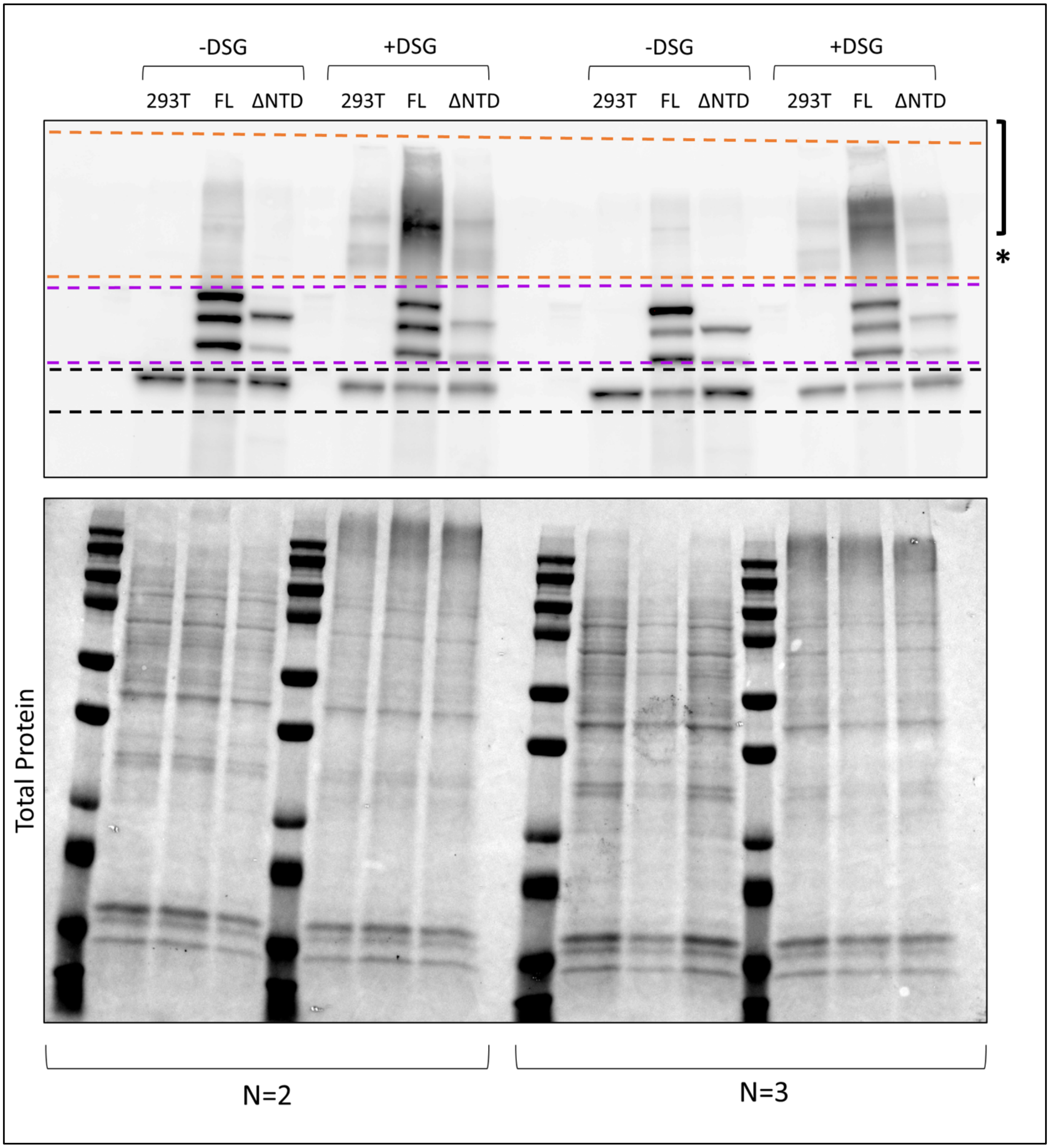

Fig. S8. Western blots of un-crosslinked and crosslinked HEK293T cells expressing FL and ΔNTD mCherry biosensors. Each blot shows an independent transfection, harvest and western blotting for TDP-43. Regions delimited by black, purple and orange dashed lines indicate endogenous monomer, biosensor monomer and cross-linked (dimer/multimer) TDP-43 species, respectively. Square brackets indicate multimeric species (>100 kDa), asterisk (*) indicates putative dimeric bands (neighboring 100 kDa).

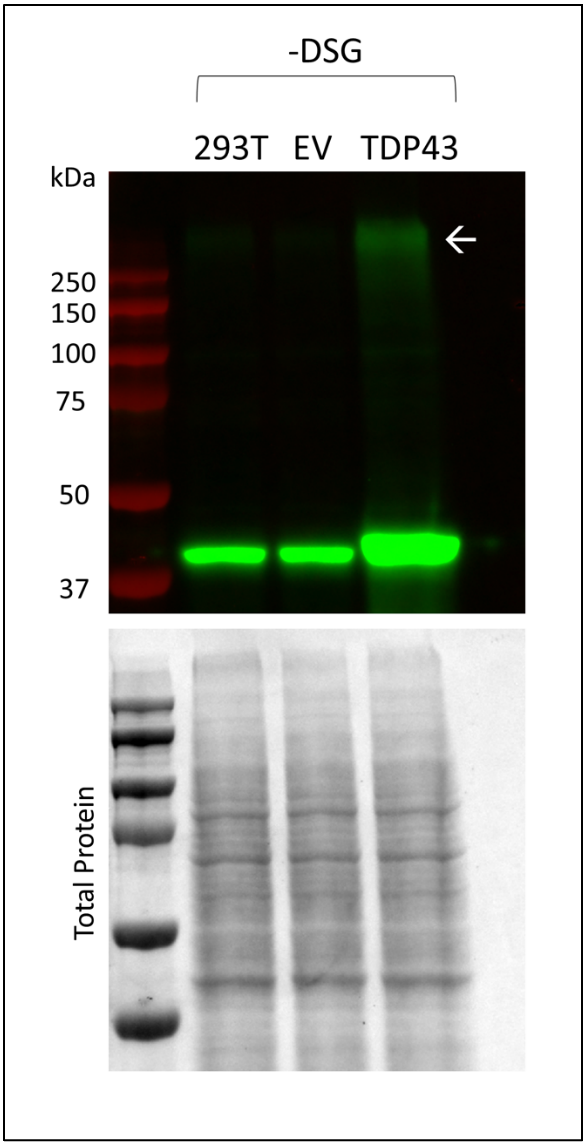

Fig. S9. Un-crosslinked western blot showing expression of TDP-43 in untransfected, empty vector and unlabeled TDP-43 transfected HEK93T cells. White arrow indicates presence of HMW band visible in unlabeled TDP-43 overexpression condition (also observed in un-crosslinked FL lanes in Fig. S5 and S8) but not untransfected or empty vector transfected cells.

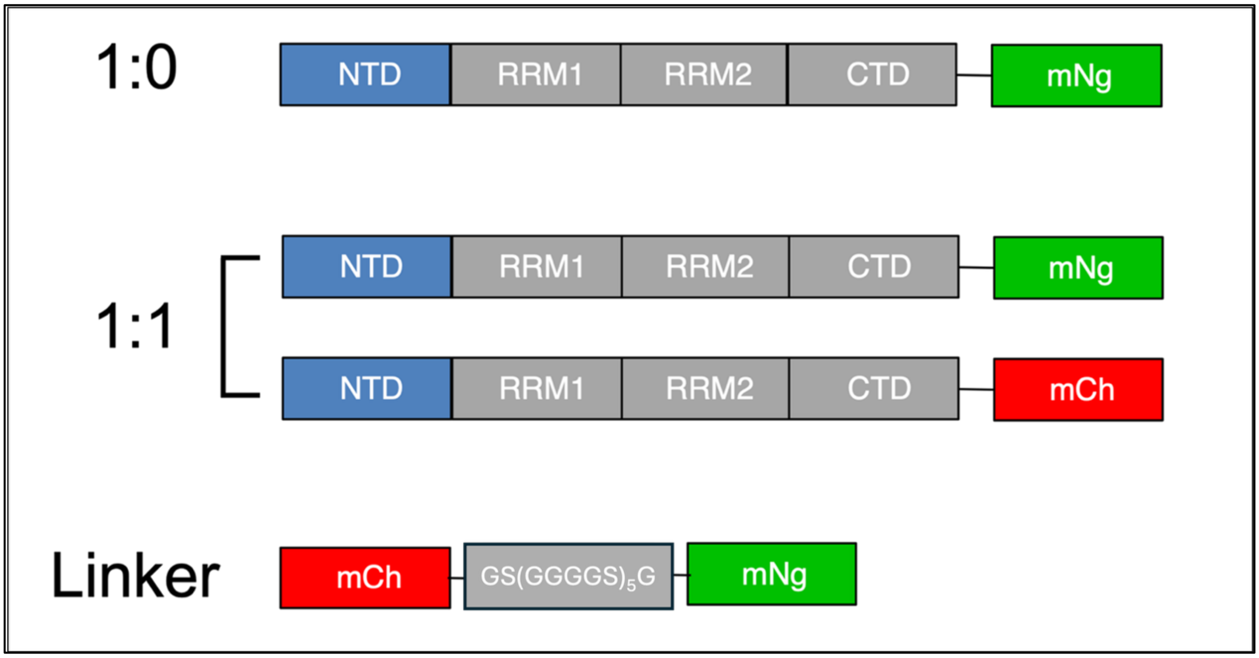

Fig. S10. Diagrams of FRET biosensors used in FDA-approved Selleck library screen.

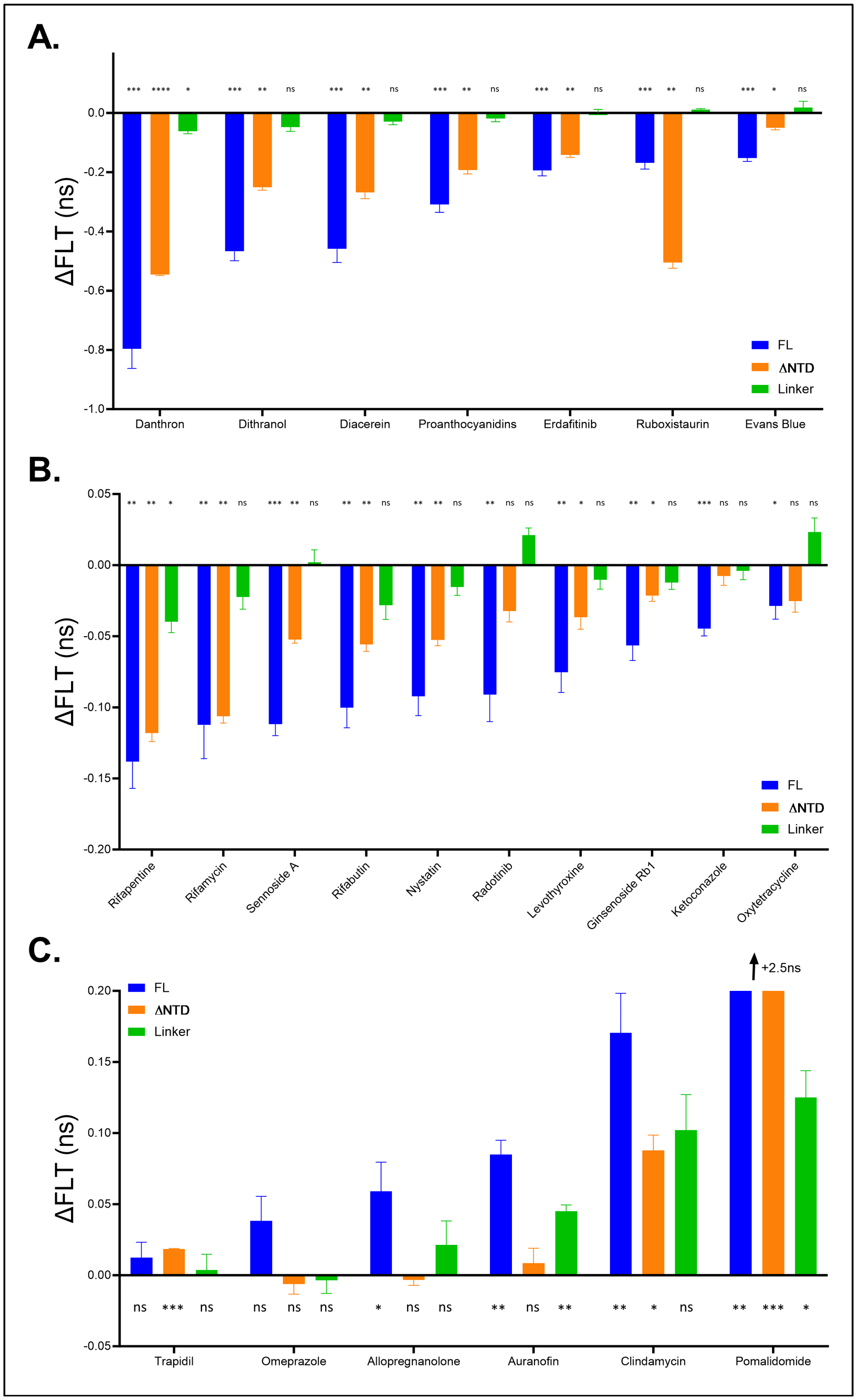

Fig. S11. ΔFLT response of all 23 TDP-43 unique hit compounds. ΔFLT ($FLT_{drug} - FLT_{DMSO}$) for hit compounds organized by effect size and direction **(A-C)**. Blue = FL TDP-43 biosensor, Orange: ΔNTD TDP-43 biosensor, Green: linker control biosensor. Statistics shown are one sample T tests to hypothetical mean of zero (DMSO treatment, * $p < 0.05$, ** $p < 0.01$, ***$p < 0.001$, **** $p < 0.0001$). Data shown are mean ± SEM from N=3 independent experiments.

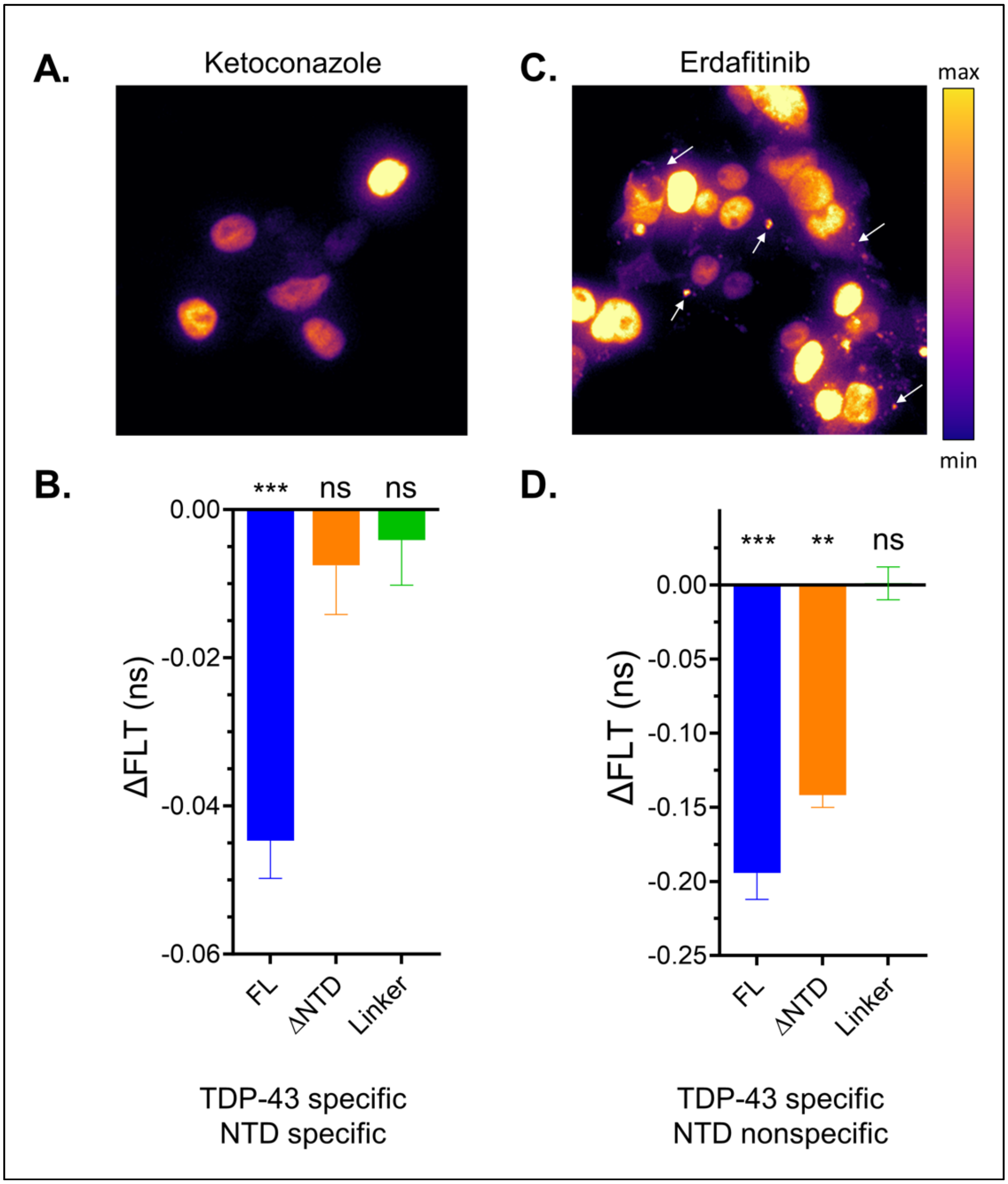

Fig. S12. NTD-specific hit ketoconazole does not induce TDP-43 aggregation. (A) Fluorescence live-cell imaging of TDP-43-mNg expressing HEK293T cells treated with 10 µM ketoconazole for 2 hours. Green fluorescence was mapped to a pseudo-color LUT. **(B)** ΔFLT profile for ketoconazole. **(C)** Fluorescence live-cell imaging of TDP-43-mNg expressing HEK293T cells treated with 10 µM erdafitinib for 2 hours. Green fluorescence was mapped to a pseudo-color LUT. White arrows indicate cytoplasmic TDP-43 puncta induced by erdafitinib. **(D)** ΔFLT profile for erdafitinib. Statistics shown are one sample T tests to hypothetical mean of zero (* $p < 0.05$, ** $p < 0.01$, ***$p < 0.001$). Data shown are mean ± SEM from N=3 independent experiments (from Fig. S11, included for ease of comparison).

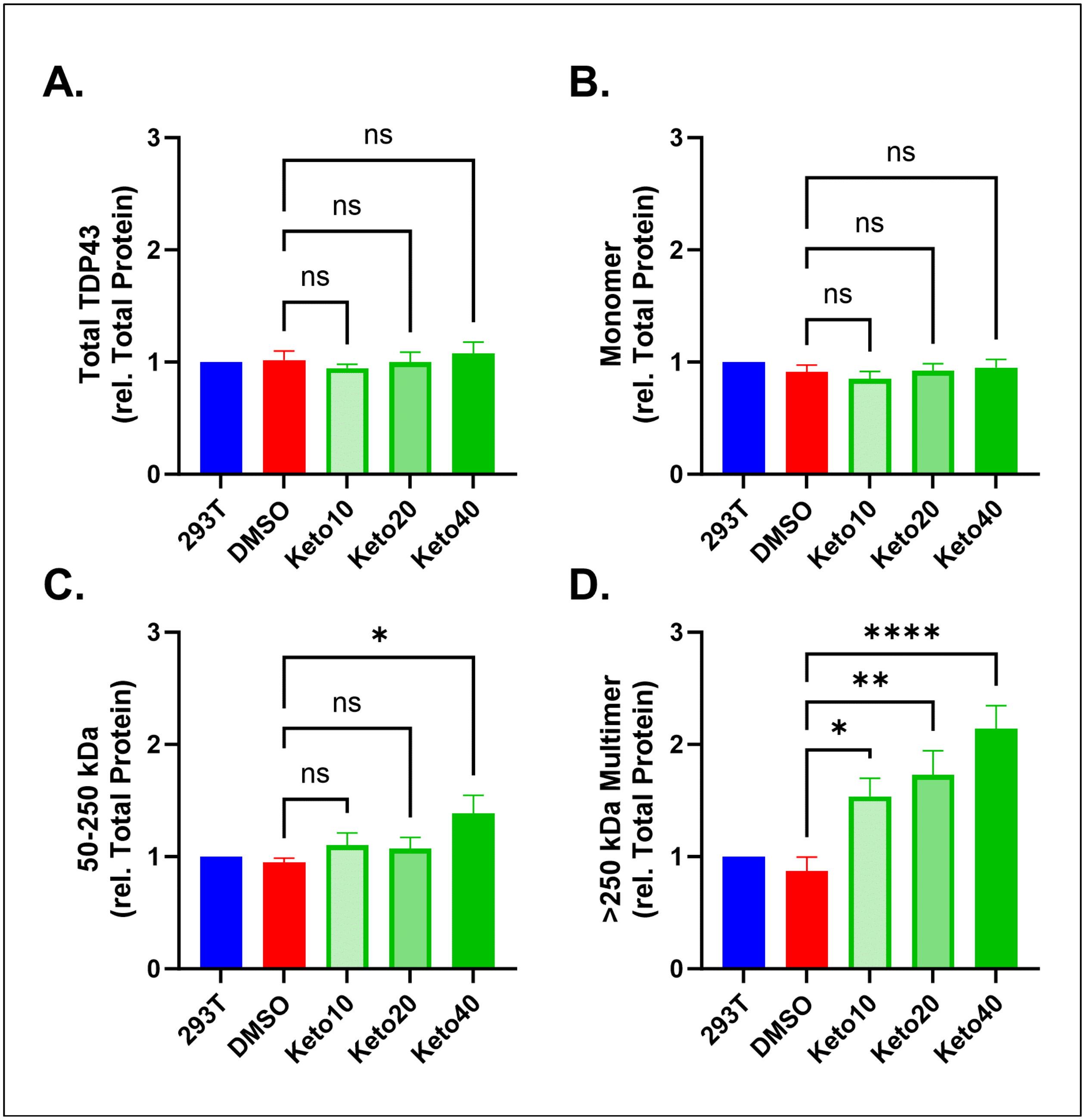

Fig. S13. Normalized levels of endogenous DSG cross-linked TDP-43 species under DMSO and ketoconazole treatment. (A) Normalized total TDP-43 levels (whole lane). **(B)** Normalized monomeric TDP-43 levels. **(C)** Normalized 50-250 kDa cross-linked TDP-43 species levels. **(D)** Normalized >250 kDa cross-linked TDP-43 species levels. Each lane was internally normalized using total protein Ponceau stain. Within each experiment, data was normalized to untreated cells (293T, blue bar shown for reference). Statistics shown are one-way ANOVAs with multiple comparisons relative to DMSO with Bonferroni correction (* $p < 0.05$, ** $p < 0.01$, *** $p < 0.001$, **** $p < 0.0001$). Data shown are mean ± SEM from N=9 independent treatments.

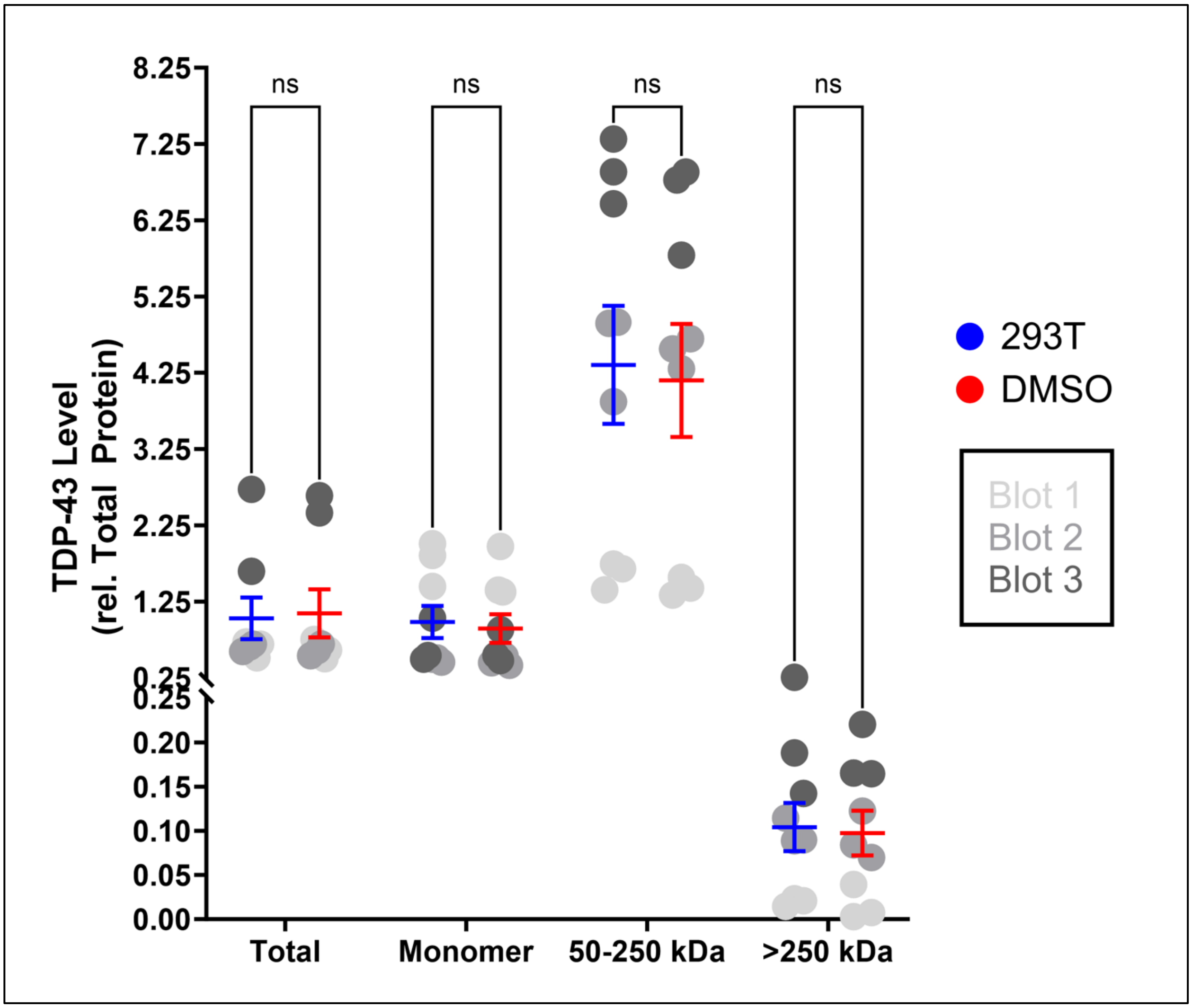

Fig. S14. Unnormalized levels of endogenous DSG cross-linked TDP-43 species in untreated and DMSO-treated cells. Statistics shown are a two-way ANOVAs with multiple comparisons relative to untreated cells (293T) with Bonferroni correction (ns, $p > 0.9999$ for all comparisons). Each data point represents a single treatment only normalized to total protein Ponceau stain, color-coded by blot # the sample was loaded into. Note the two different y-axis segments. Data shown (blue and red lines) are mean ± SEM from N=9 independent treatments.

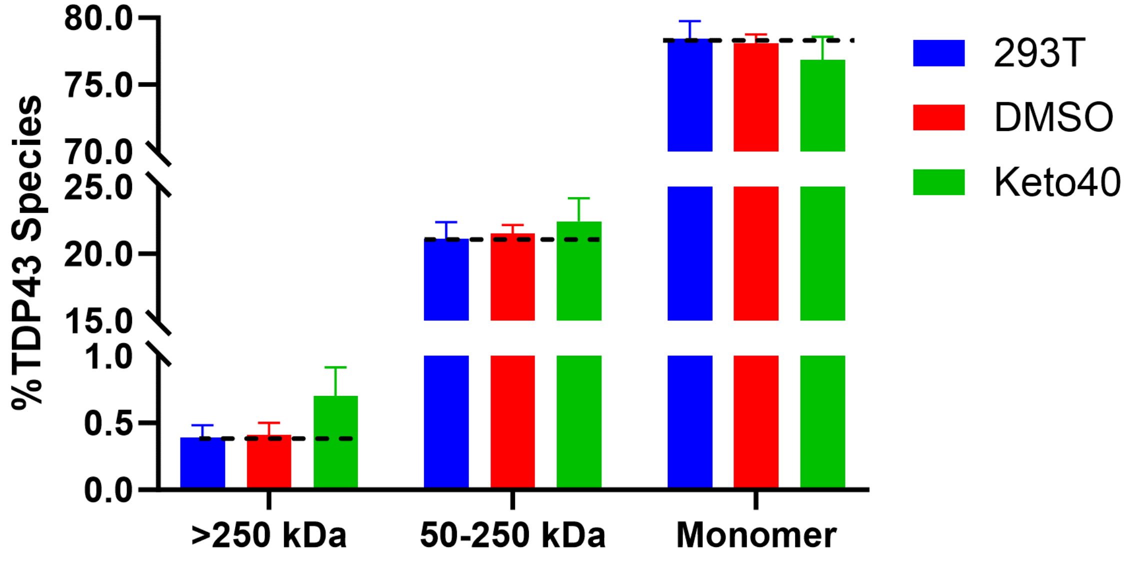

Fig. S15. Relative amounts of endogenous TDP-43 cross-linked species under DMSO and 40 µM ketoconazole treatment. Note the three different segments in the y-axis. No comparisons to DMSO are statistically significant. Black dashed line indicates untreated (293T) average value.

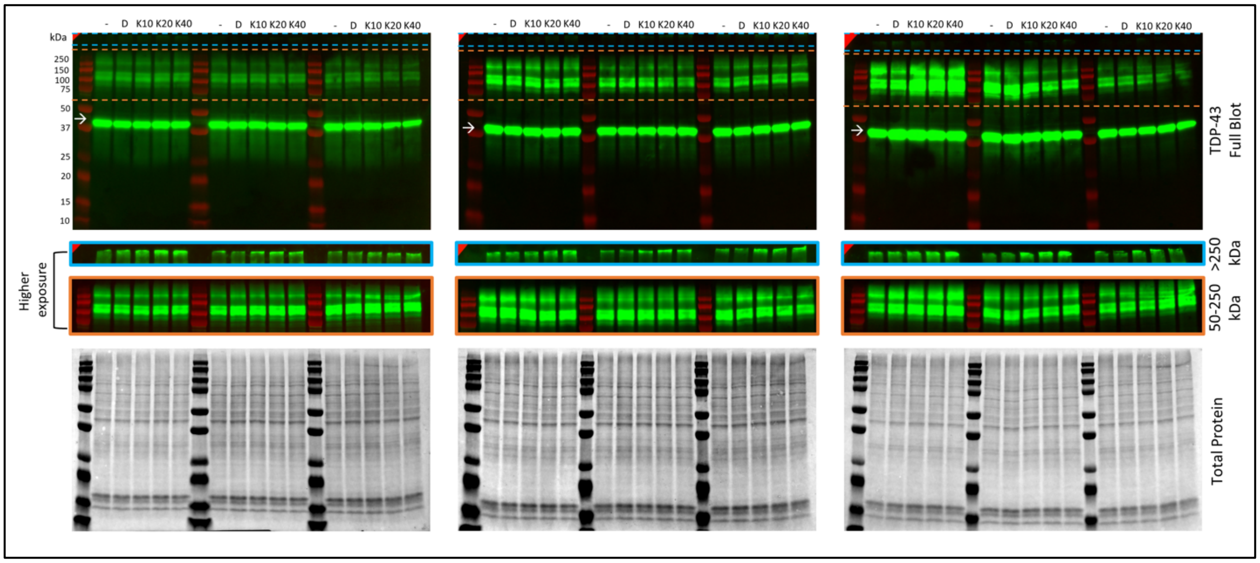

Fig. S16. Western blots of endogenous DSG cross-linked TDP-43 species under DMSO and ketoconazole treatment. N=9 treatments were imaged in three separate blots. Blue dashed lines indicate >250 kDa cross-linked species region shown below full blot. Orange dashed lines indicate 50-250 kDa cross-linked species region shown below full blot. White arrow (→) indicates monomeric TDP-43.

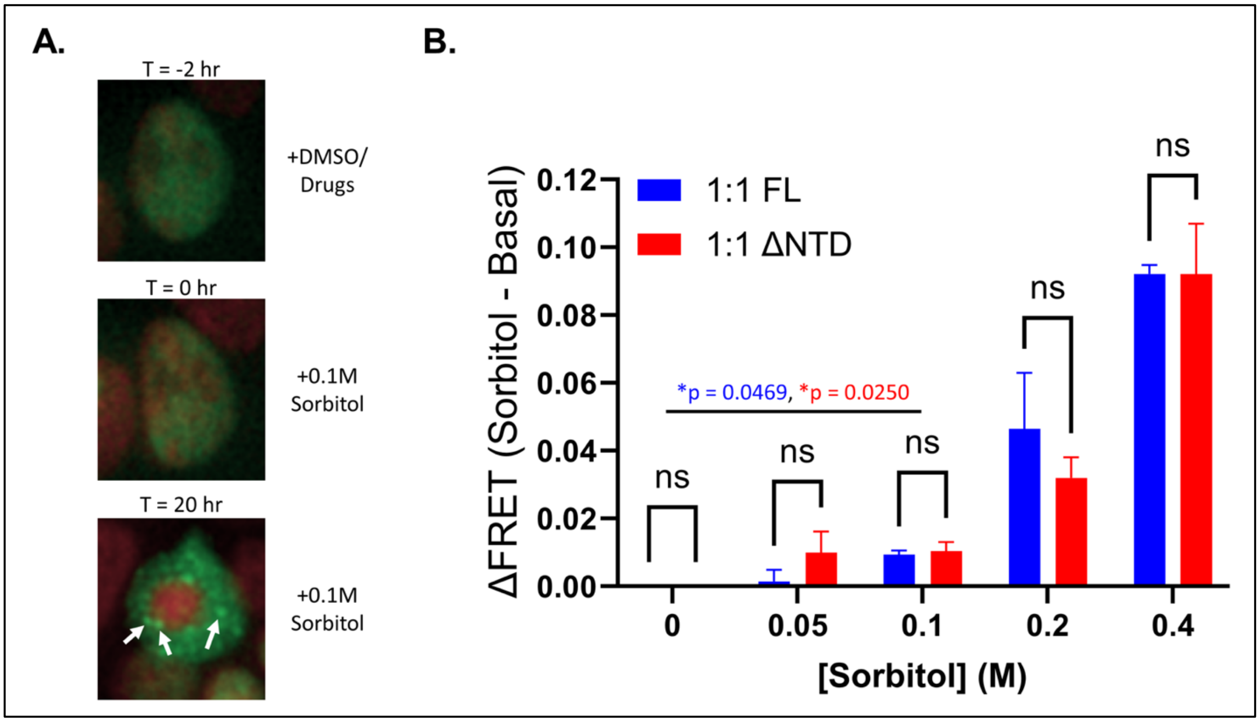

Fig. S17. Sorbitol effect on TDP-43 FRET and subcellular localization. (A) Representative image of TDP43-mNg expressing cell treated with 0.1 M sorbitol for 20 hours. White arrows indicate TDP-43 puncta induced by sorbitol treatment. TDP-43-mNg shown in green and nuclear stain (Hoechst) shown in red pseudo-color. **(B)** Sorbitol titration effect on full-length and ΔNTD TDP-43 biosensor FRET. Statistics shown are two-way ANOVA multiple comparisons with Bonferroni correction (between full-length and ΔNTD bars at single sorbitol concentration) and unpaired T-test (between 0 M and 0.1 M sorbitol for each biosensor, $*p < 0.05$). Data shown are mean ± SEM from N=3 independent experiments.

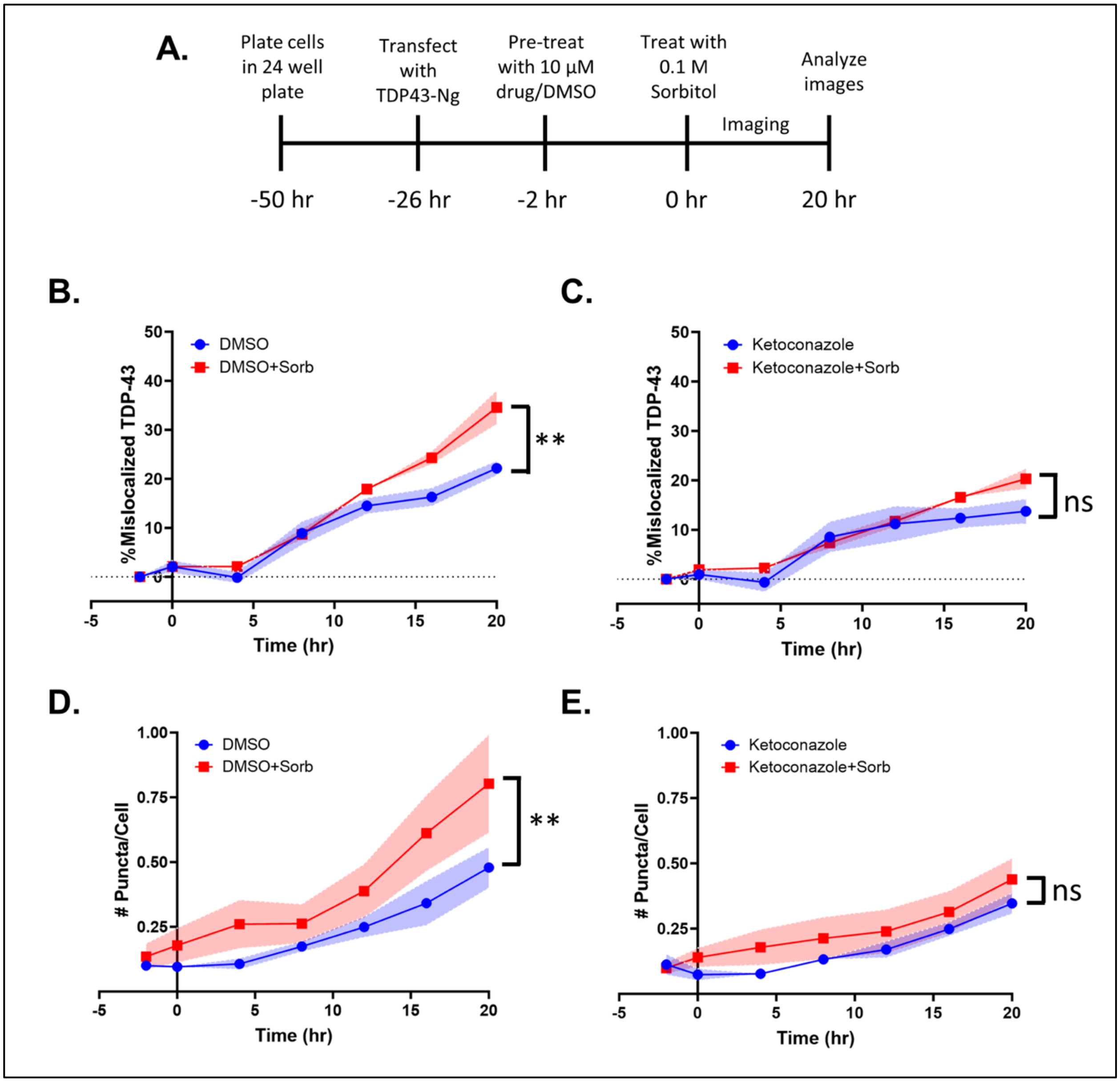

Fig. S18. Sorbitol-induced TDP-43 puncta formation and mislocalization experimental design and traces. (A) Experimental treatment timeline. **(B)** Average TDP-43 mislocalization -/+ sorbitol under DMSO treatment. **(C)** Average TDP-43 mislocalization -/+ sorbitol under ketoconazole treatment. **(D)** Average TDP-43 puncta formation -/+ sorbitol under DMSO treatment. **(E)** Average TDP-43 puncta formation -/+ sorbitol under ketoconazole treatment. Data shown are mean ± SEM from N=3 independent imaging experiments. Statistics shown are two-way ANOVA multiple comparisons with Bonferroni correction (*p $<$ 0.05, **p<0.01, ***p<0.001).

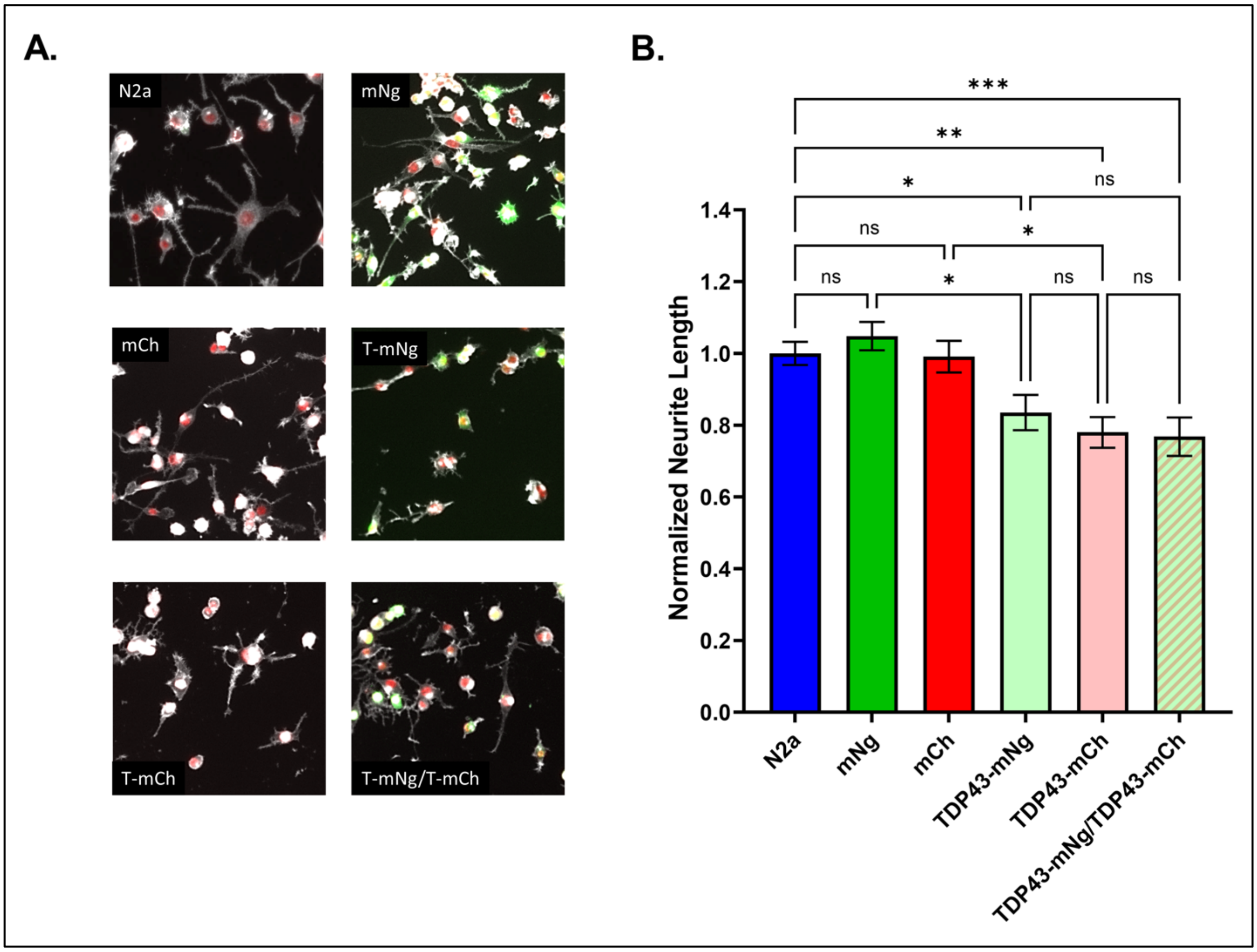

Fig. S19. Neurite growth assay in retinoic acid differentiated normal and transiently transfected N2a cells. (A) Representative images of differentiated untransfected and mNg, mCh, TDP-43-mNg, TDP-43-mCh and TDP-43-mNg/TDP-43-mCh (1:1) transfected N2a's. Channels shown are phalloidin rhodamine actin stain and mCherry (gray pseudo-colored), nuclear Hoechst stain (red pseudo-color) and mNeonGreen (green). **(B)** Normalized neurite length (total neurite length normalized to number of cells in each image) for each condition. Statistics shown are one-way ANOVA multiple comparisons adjusted with Bonferroni correction. Data shown are mean ± SEM from N=3 independent experiments with at least 6 images per condition for each run.

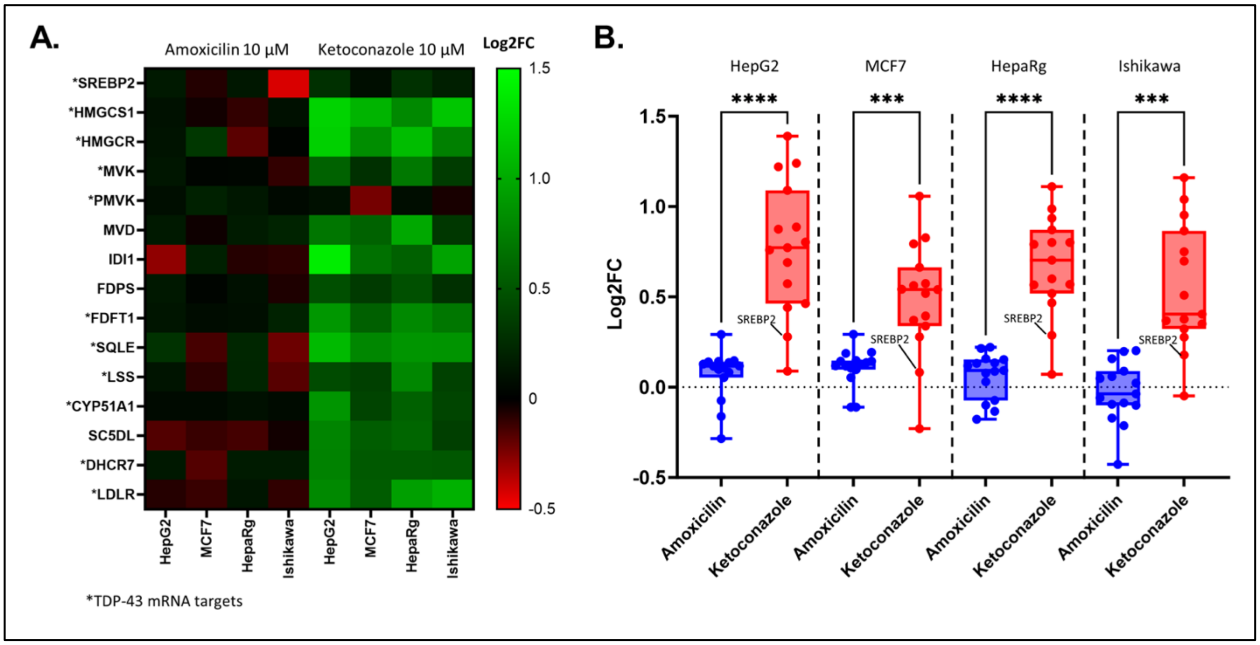

Fig. S20. Transcriptomics mining of 10 µM ketoconazole effect on cholesterol biosynthesis genes in 4 different immortalized cell lines. (A) Log2FC (relative to vehicle) heatmap of SREBP2 cholesterol synthesis regulated genes for 6-hour 10 µM amoxicillin (non-hit in FLT-FRET screen) and 10 µM ketoconazole (FLT-FRET hit) in HepG2, MCF7, HepaRg and Ishikawa cells. Asterisks (*) indicate genes known to be mRNA binding targets of TDP-43 protein. **(B)** Summary of Log2FC for genes shown in (A). Statistics shown are one-way ANOVA multiple comparisons with Bonferroni correction (***$p < 0.001$, ****$p < 0.0001$). Data shown are mean from N=3 independent sample treatments. Data presented here was mined from De Abrew et. al. public dataset (40).

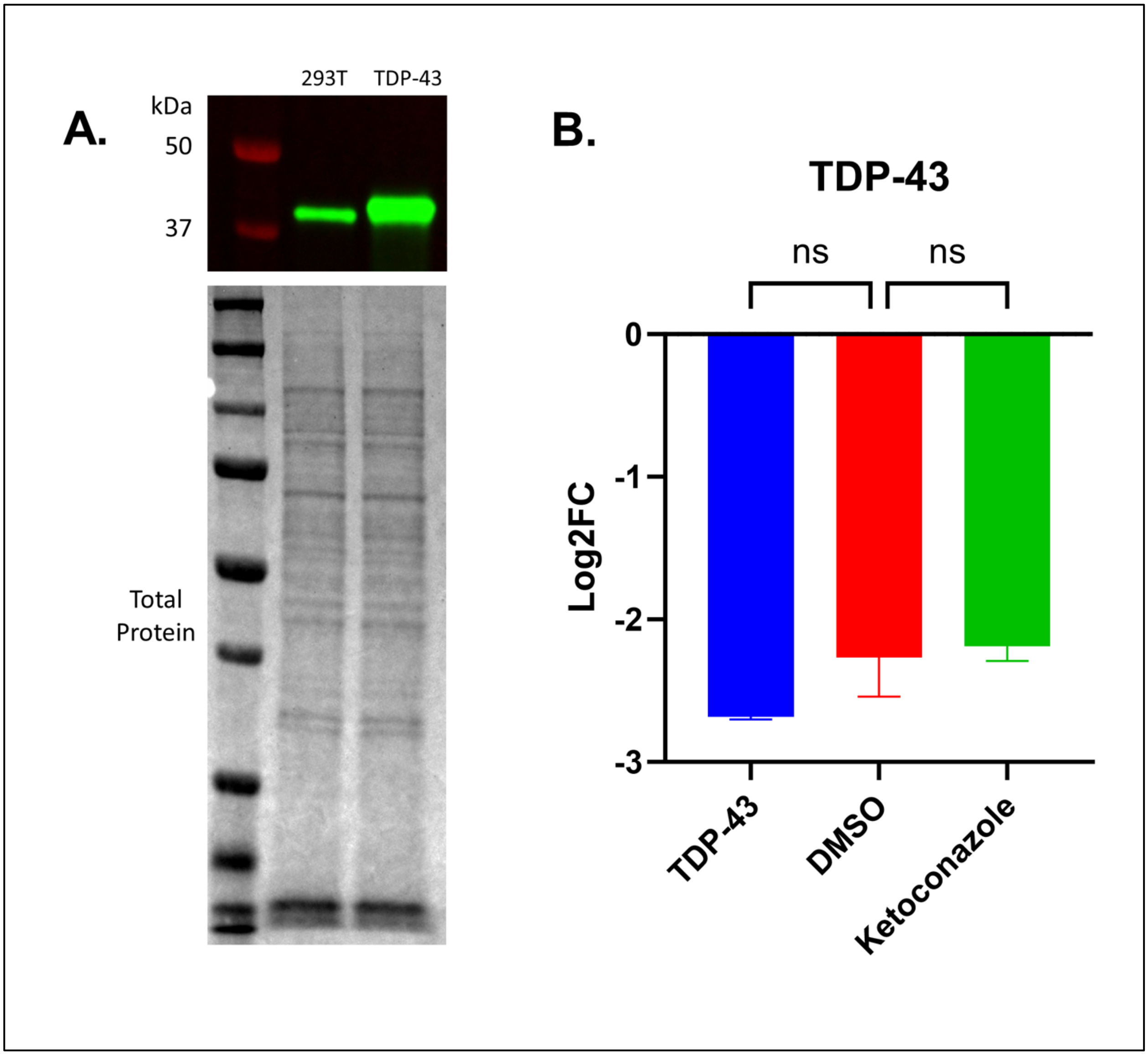

Fig. S21. RT-qPCR assay probing for endogenous TDP-43 under TDP-43 overexpression. (A) Unlabeled TDP-43 overexpression confirmation in HEK293T cells via western blot. **(B)** Log2FC of endogenous TDP-43 mRNA in untreated, DMSO-treated or ketoconazole-treated HEK293T cells overexpressing TDP-43. Log2FC values were calculated relative to non-transfected HEK293T cells and using GAPDH as a housekeeping gene. Statistics shown are one-way ANOVA multiple comparisons with Bonferroni correction against DMSO-treated TDP-43 overexpressing cells (red bar, $**p < 0.01$). Data shown are mean ± SEM from N=3 independent experiments.

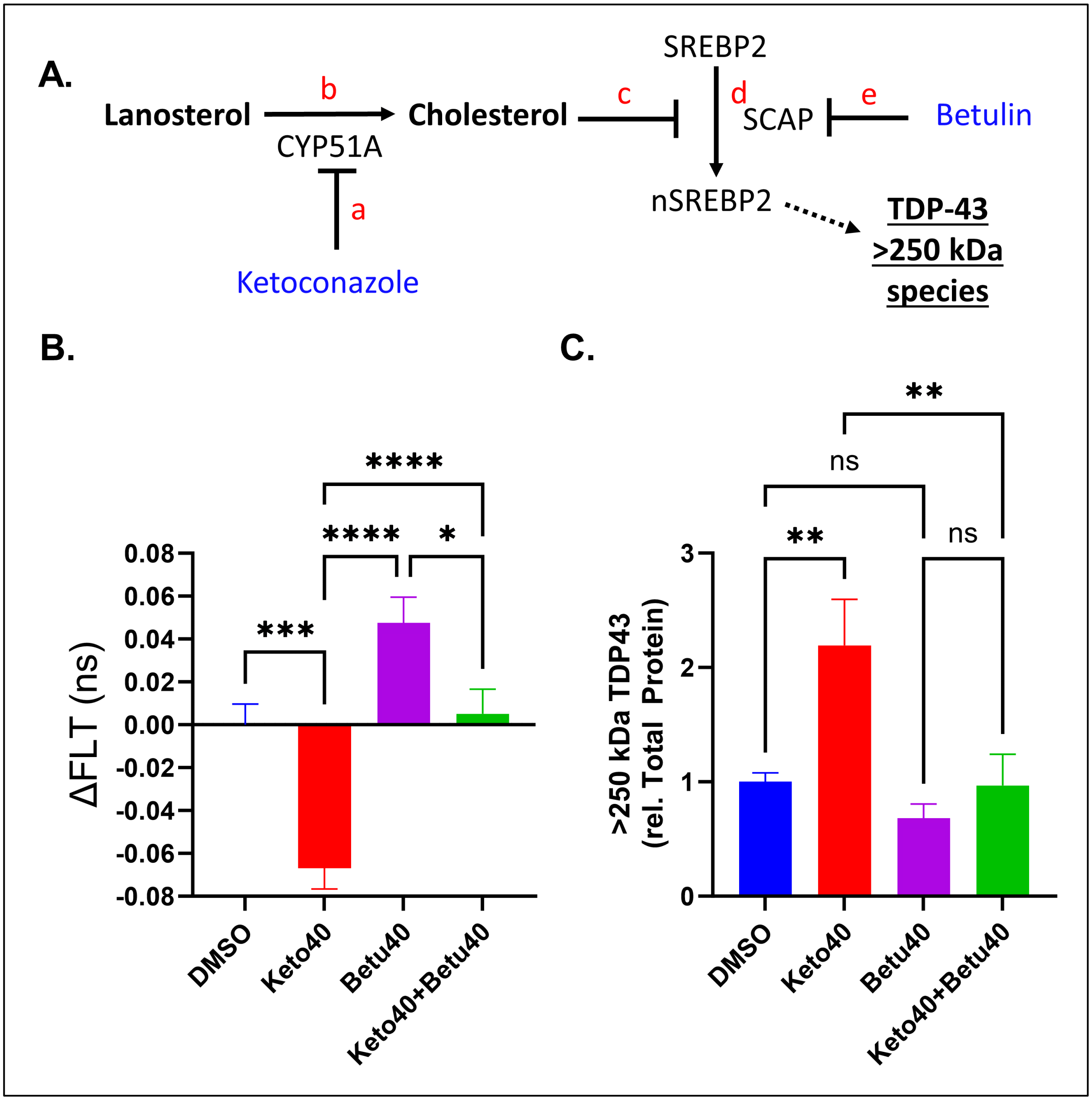

Fig. S22. Betulin counteracts the increase in high-molecular weight TDP-43 species induced by ketoconazole. (A) Proposed mechanism of action schematic for ketoconazole and betulin. Solid lines with red letters indicate proposed mechanistic steps experimentally validated in the literature but not this study (a: Strushkevich et. al. (*3*), b: Zhu et. al. (*4*), c: DeBose-Boyd et. al. (*5*), d: Lee et. al. (*6*), e: Tang et. al. (*7*)). Dashed line indicates proposed mechanistic step not experimentally validated in the literature nor this study. **(B)** ΔFLT ($FLT_{drug} - FLT_{DMSO}$) induced by 40 µM ketoconazole, 40 µM betulin and co-treatment. Data shown are mean ± SEM from at least N=7 independent treatments. **(C)** Normalized levels of >250 kDa endogenous TPD-43 cross-linked species under 40 µM ketoconazole, 40 µM betulin and co-treatment. Data shown are mean ± SEM from at least N=6 independent treatments. Statistics shown are one-way ANOVA multiple comparisons with Bonferroni correction (*$p < 0.05$, ***$p < 0.001$, ****$p < 0.0001$).

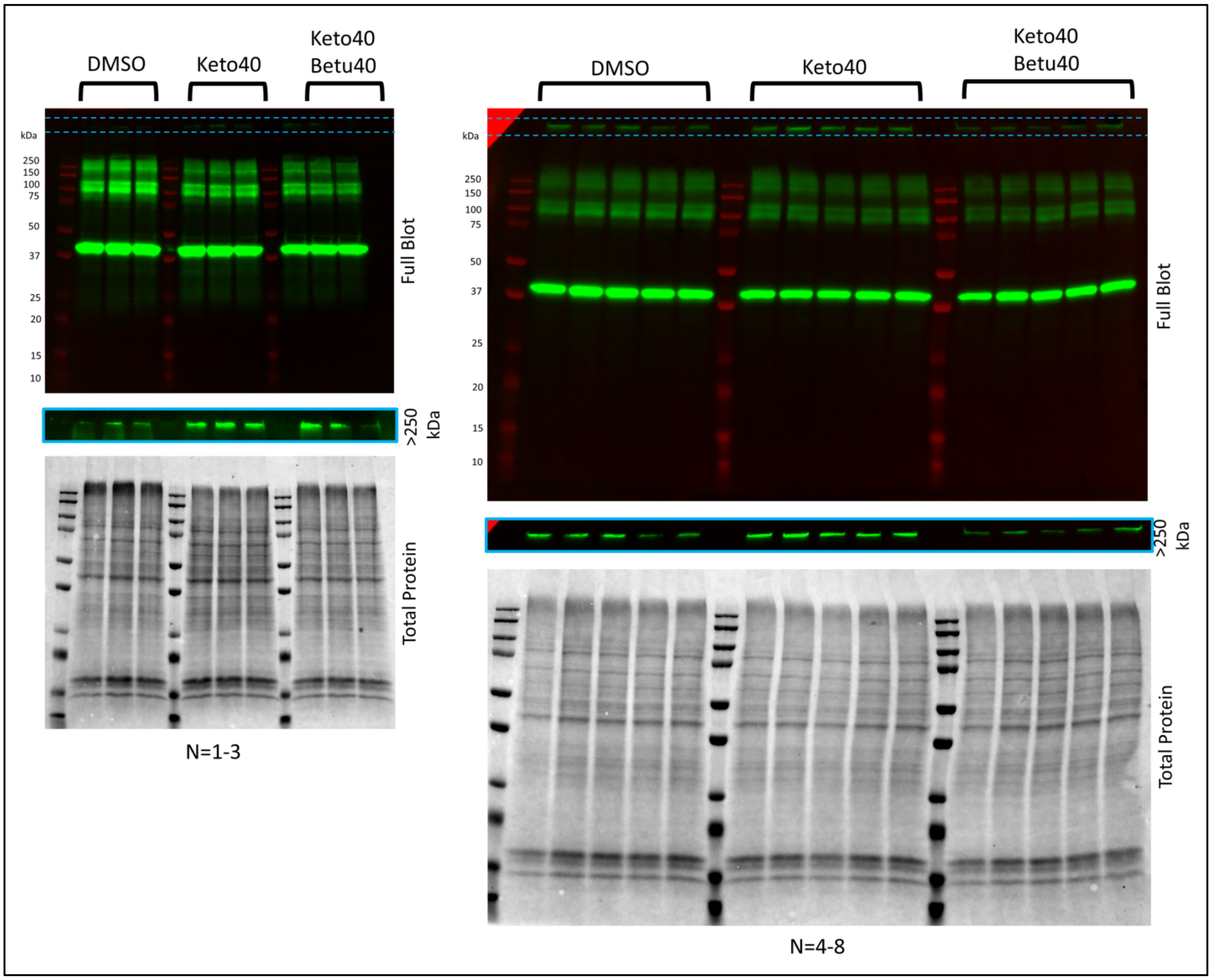

Fig. S23. Western blots of endogenous DSG cross-linked TDP-43 species under DMSO, ketoconazole and betulin treatments. N=8 treatments were imaged in two separate blots. Blue dashed lines indicate >250 kDa cross-linked species region shown below full blot.

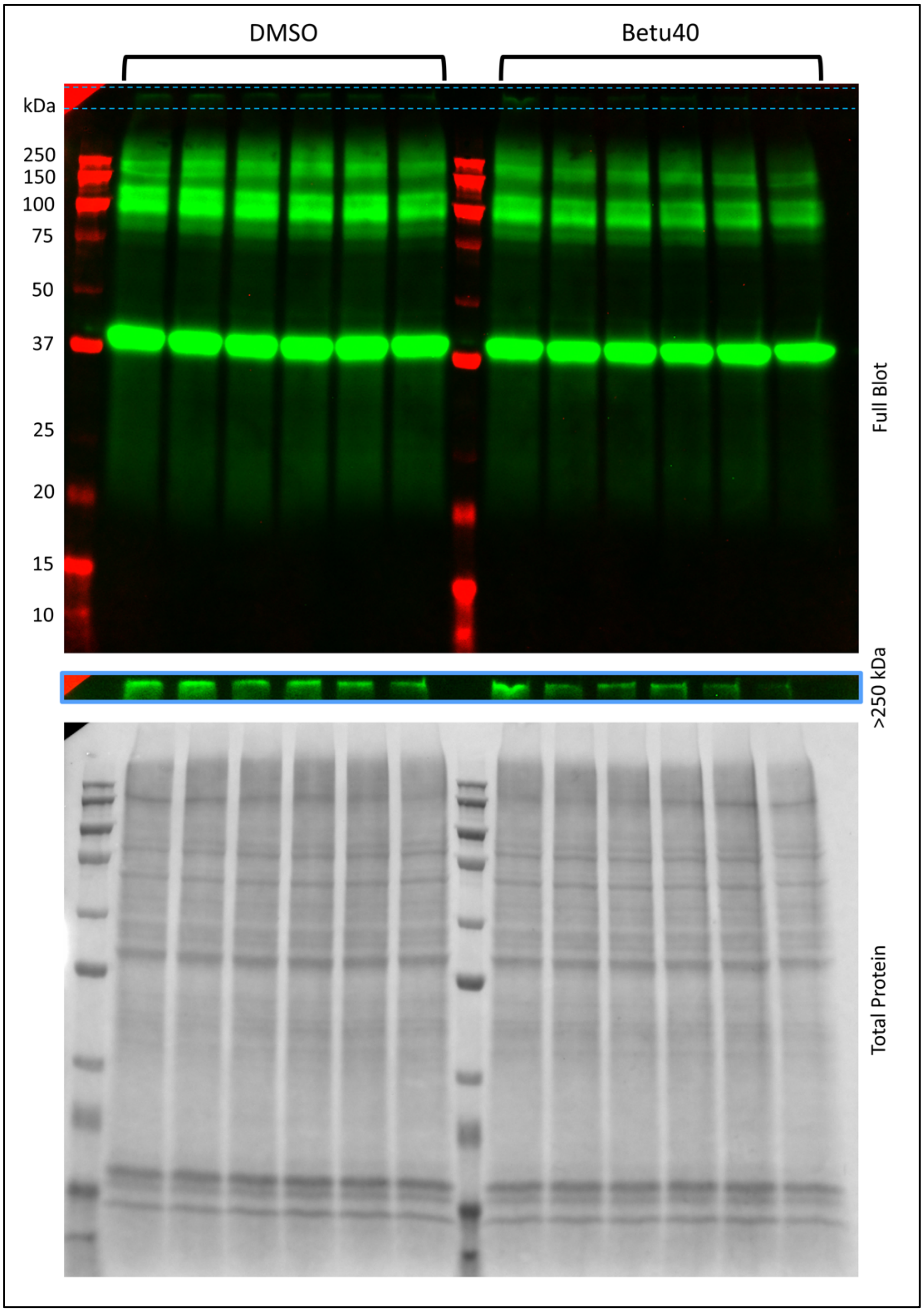

Fig. S24. Western blot of endogenous DSG cross-linked TDP-43 species under DMSO and betulin-only treatments. N=6 treatments were imaged. Blue dashed lines indicate >250 kDa cross-linked species region shown below full blot.

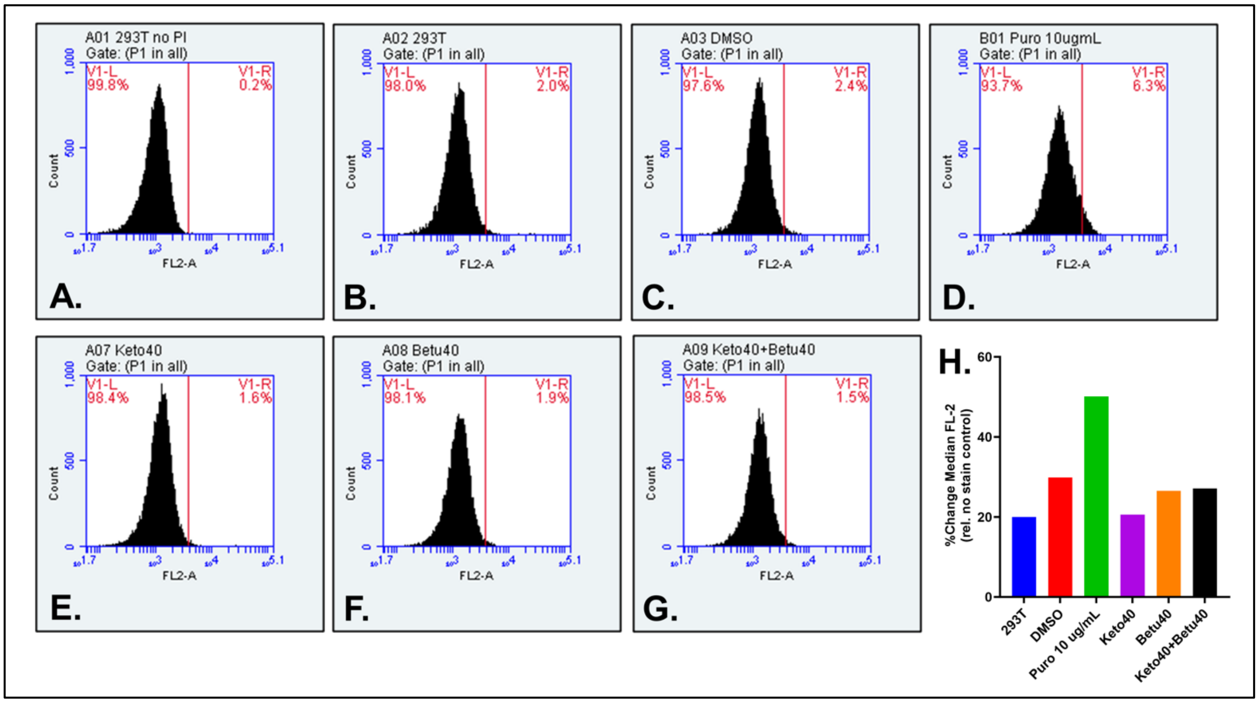

Fig. S25. Propidium iodide staining flow cytometry of ketoconazole and betulin treated HEK293T cells. FL2 histograms for **(A)** unstained untreated, **(B)** stained untreated, **(C)** DMSO, **(D)** 10 µg/mL puromycin (positive control), **(E)** 40 µM ketoconazole, **(F)** 40 µM betulin and **(G)** 40 µM ketoconazole + 40 µM betulin. **(H)** Percent change in median FL2 intensity for conditions (B-G), relative to unstained untreated cells.

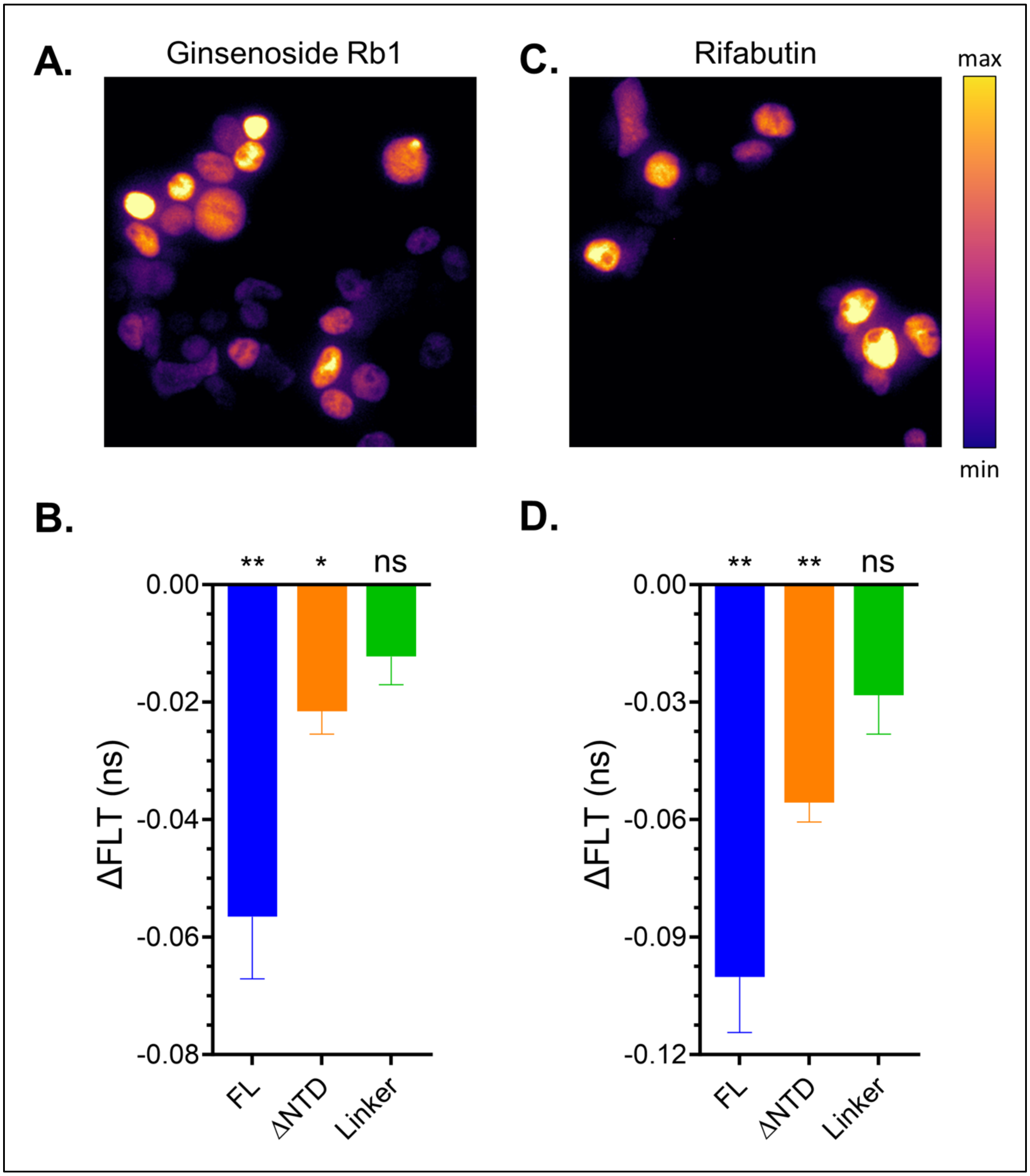

Fig. S26. Partial NTD-specific hits do not induce TDP-43 aggregation. (A) Fluorescence live-cell imaging of TDP-43-mNg expressing HEK293T cells treated with 10 μM ginsenoside Rb1 for 2 hours. Green fluorescence was mapped to a pseudo-color LUT. **(B)** ΔFLT profile for ginsenoside Rb1. **(C)** Fluorescence live-cell imaging of TDP-43-mNg expressing HEK293T cells treated with 10 μM rifabutin for 2 hours. Green fluorescence was mapped to a pseudo-color LUT. **(D)** ΔFLT profile for rifabutin. Statistics shown are one sample T tests to hypothetical mean of zero (DMSO treatment, * $p < 0.05$, ** $p < 0.01$). Data shown are mean ± SEM from N=3 independent experiments (from Fig. S11, included for ease of comparison).

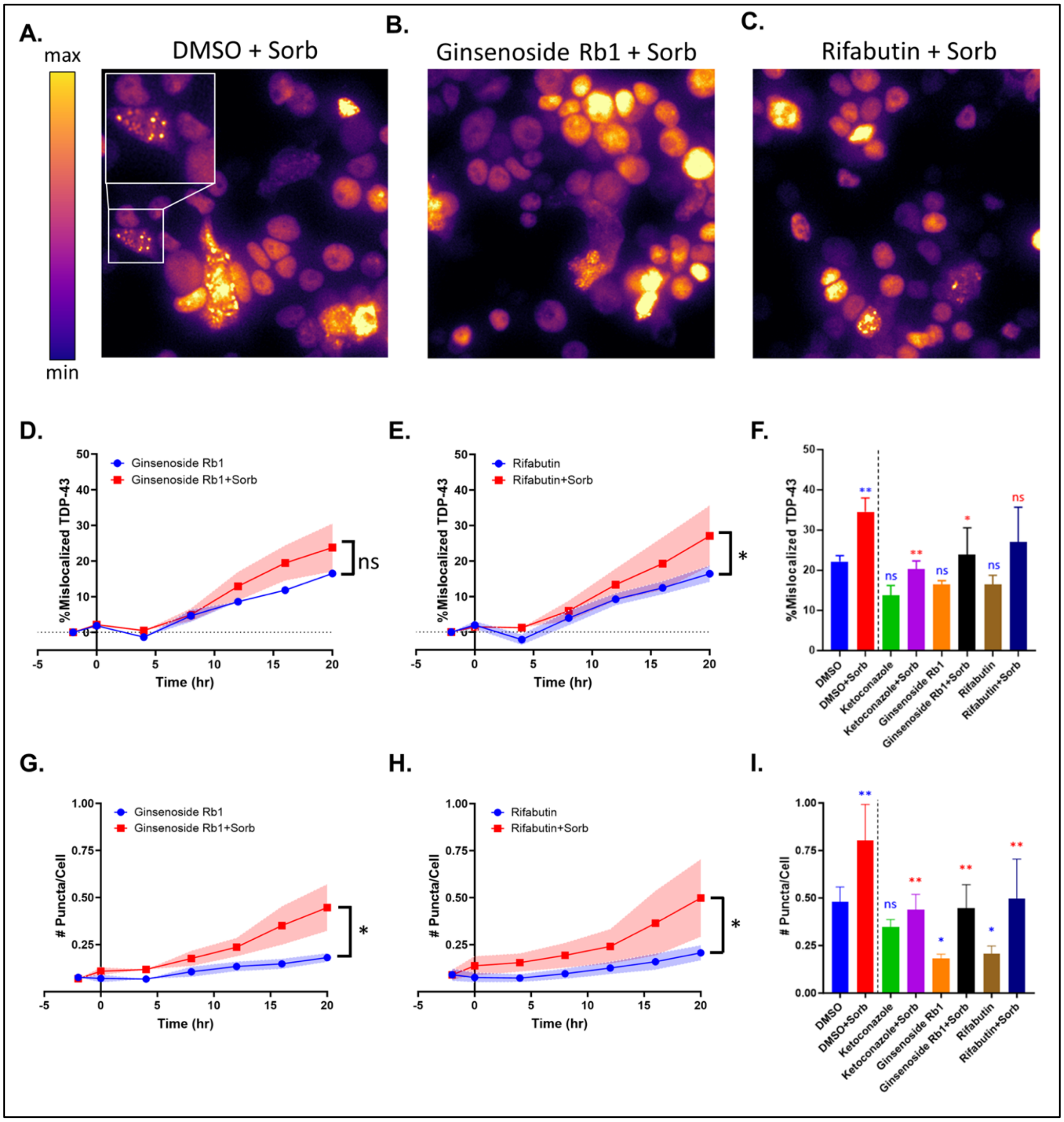

Fig. S27. Sorbitol-induced TDP-43 puncta formation and mislocalization under ginsenoside Rb1 and rifabutin treatment. Fluorescence live cell imaging of HEK293T cells expressing FL TDP-43-mNg treated with 0.1 M sorbitol under **(A)** DMSO, **(B)** 10 µM ginsenoside Rb1 or **(C)** 10 µM rifabutin. Green fluorescence was mapped to a pseudo-color LUT. **(D)** Average TDP-43 mislocalization -/+ sorbitol under ginsenoside Rb1 treatment. **(E)** Average TDP-43 mislocalization -/+ sorbitol under rifabutin treatment. **(F)** Endpoint quantification of TDP-43 mislocalization for all treatments (ketoconazole included for reference). **(G)** Average TDP-43 puncta -/+ sorbitol under ginsenoside Rb1 treatment. **(H)** Average TDP-43 puncta -/+ sorbitol under rifabutin treatment. **(I)** Endpoint quantification of TDP-43 puncta for all treatments (ketoconazole included for reference). Data shown are mean ± SEM from N=3 independent imaging experiments. Statistics shown are two-way ANOVA multiple comparisons with Bonferroni corrections for panels (D), (E), (G) and (H). In panels (F) and (I), blue indicates statistics relative to DMSO-only and red indicates statistics relative to DMSO+Sorbitol (*$p < 0.05$, **$p < 0.01$, ***$p < 0.001$).

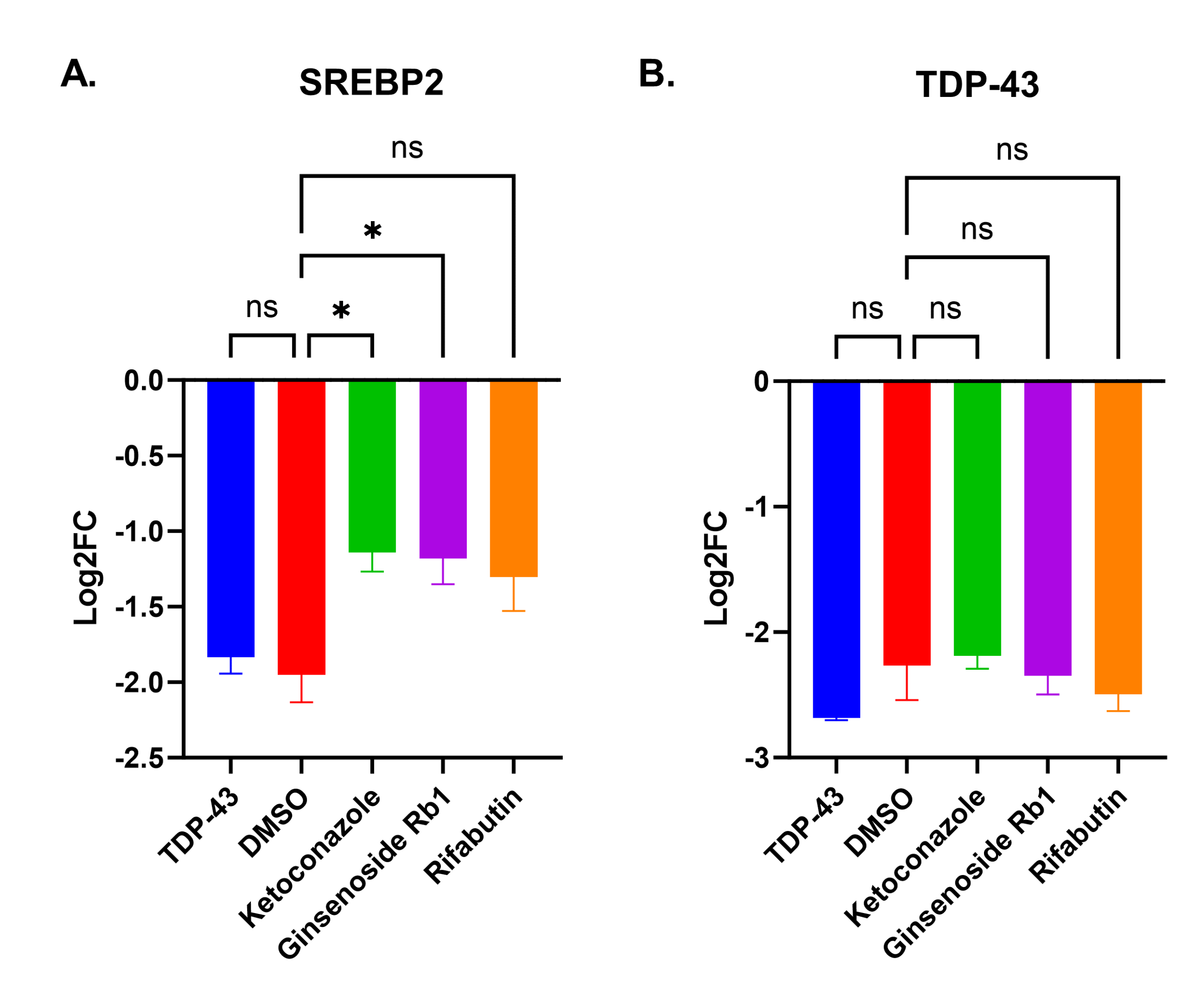

Fig. S28. RT-qPCR assay probing for endogenous SREBP2 and TDP-43 under TDP-43 overexpression. (A) Log2FCof endogenous TDP-43 mRNA in untreated, DMSO-treated or 10 µM drug-treated HEK293T cells overexpressing TDP-43. **(B)** Log2FC of endogenous SREBP2 mRNA in untreated, DMSO-treated or drug-treated HEK293T cells overexpressing TDP-43. Ketoconazole data included for reference. Log2FC values were calculated relative to untransfected/untreated HEK293T cells and using GAPDH as a housekeeping gene. Statistics shown are one-way ANOVA multiple comparisons with Bonferroni correction against untreated TDP-43 overexpressing cells (red bar, $*p < 0.05$). Data shown are mean ± SEM from N=3 independent experiments.

Movie S1: Representative swimming assay recording of untreated control (OW1603) C. elegans.

Movie S2: Representative swimming assay recording of DMSO-treated control (OW1603) C. elegans.

Movie S3: Representative swimming assay recording of 10 μM ketoconazole treated control (OW1603) C. elegans.

Movie S4: Representative swimming assay recording of untreated TDP-43 (OW1601) C. elegans.

Movie S5: Representative swimming assay recording of DMSO-treated TDP-43 (OW1601) C. elegans.

Movie S6: Representative swimming assay recording of 10 μM ketoconazole treated TDP-43 (OW1601) C. elegans.

References

1. L. Huang, D. Pike, D. E. Sleat, V. Nanda, P. Lobel, Potential pitfalls and solutions for use of fluorescent fusion proteins to study the lysosome. *PLoS ONE* **9**, (2014).
2. M. Pérez-Berlanga, V. I. Wiersma, A. Zbinden, L. De Vos, U. Wagner, C. Foglieni, I. Mallona, K. M. Betz, A. Cléry, J. Weber, Loss of TDP-43 oligomerization or RNA binding elicits distinct aggregation patterns. *The EMBO journal* **42**, e111719 (2023).
3. N. Strushkevich, S. A. Usanov, H.-W. Park, Structural Basis of Human CYP51 Inhibition by Antifungal Azoles. *Journal of Molecular Biology* **397**, 1067-1078 (2010).
4. J. Zhu, K. Mounzih, E. F. Chehab, N. Mitro, E. Saez, F. F. Chehab, Effects of FoxO4 overexpression on cholesterol biosynthesis, triacylglycerol accumulation, and glucose uptake. *Journal of Lipid Research* **51**, 1312-1324 (2010).
5. R. A. DeBose-Boyd, Feedback regulation of cholesterol synthesis: sterol-accelerated ubiquitination and degradation of HMG CoA reductase. *Nature Cell Research* **18**, 609-621 (2008).
6. S. H. Lee, J.-H. Lee, S.-S. Im, The cellular function of SCAP in metabolic signaling. *Experimental & Molecular Medicine* **52**, 724-729 (2020).
7. J.-J. Tang, J.-G. Li, W. Qi, W.-W. Qiu, P.-S. Li, B.-L. Li, B.-L. Song, Inhibition of SREBP by a Small Molecule, Betulin, Improves Hyperlipidemia and Insulin Resistance and Reduces Atherosclerotic Plaques. *Cell Metabolism* **13**, 44-56 (2011).
